# The Tommy’s Clinical Decision Tool, a device for reducing the clinical impact of placental dysfunction and preterm birth: protocol for a mixed-methods early implementation evaluation study

**DOI:** 10.1186/s12884-022-04867-w

**Published:** 2022-08-15

**Authors:** Jenny Carter, Dilly Anumba, Lia Brigante, Christy Burden, Tim Draycott, Siobhán Gillespie, Birte Harlev-Lam, Andrew Judge, Erik Lenguerrand, Elaine Sheehan, Basky Thilaganathan, Hannah Wilson, Cathy Winter, Maria Viner, Jane Sandall

**Affiliations:** 1 Department of Women and Children’s Health, School of Life Course and Population Sciences, King’s College London, 10th Floor, North Wing, St Thomas’ Hospital, Westminster Bridge Road, London, SE1 7EH UK; 2 Tommy’s National Centre for Maternity Improvement, Royal College of Obstetricians and Gynaecologists/Royal College of Midwives, 10-18 Union Street, London, SE1 1SZ UK; 3 Department of Oncology and Metabolism, University of Sheffield, The Jessop Wing, Tree Root Walk, Sheffield, S10 2SF UK; 4 Royal College of Midwives, 10-18 Union Street, London, SE1 1SZ UK; 5 Academic Women’s Health Unit, University of Bristol, Bristol Medical School, Southmead Hospital, Bristol, BS10 5NB UK; 6 Royal College of Obstetricians and Gynaecologists, 10-18 Union Street, London, SE1 1SZ UK; 7 Translational Health Sciences, University of Bristol, Bristol Medical School, Southmead Hospital, Bristol, BS10 5NB UK; 8 Maternal Medicine Department, St George’s University Hospitals NHS Foundation Trust, Blackshaw Road, London, SW17 0QT UK; 9 Vascular Biology Research Centre, Molecular and Clinical Sciences Research Institute, St George’s University of London, Cranmer Terrace, London, SW17 0QT UK; 10 Fetal Medicine Unit, St George’s University Hospitals NHS Foundation Trust, Blackshaw Road, London, SW17 0QT UK; 11 PROMPT Maternity Foundation, Department of Women’s Health, The Chilterns, Southmead Hospital, Bristol, BS10 5NB UK; 12 Mothers for Mothers, New Fulford Family Centre, Gatehouse Avenue, Bristol, BS13 9AQ UK

**Keywords:** Decision support, Implementation, Stillbirth, Preterm, eHealth, Process evaluation, Pregnancy, Risk assessment

## Abstract

**Background:**

Disparities in stillbirth and preterm birth persist even after correction for ethnicity and social deprivation, demonstrating that there is wide geographical variation in the quality of care. To address this inequity, Tommy’s National Centre for Maternity Improvement developed the Tommy’s Clinical Decision Tool, which aims to support the provision of “the right care at the right time”, personalising risk assessment and care according to best evidence. This web-based clinical decision tool assesses the risk of preterm birth and placental dysfunction more accurately than current methods, and recommends best evidenced-based care pathways in a format accessible to both women and healthcare professionals. It also provides links to reliable sources of pregnancy information for women. The aim of this study is to evaluate implementation of Tommy’s Clinical Decision Tool in four early-adopter UK maternity services, to inform wider scale-up.

**Methods:**

The Tommy’s Clinical Decision Tool has been developed involving maternity service users and healthcare professionals in partnership. This mixed-methods study will evaluate: maternity service user and provider acceptability and experience; barriers and facilitators to implementation; reach (whether particular groups are excluded and why), fidelity (degree to which the intervention is delivered as intended), and unintended consequences. Data will be gathered over 25 months through interviews, focus groups, questionnaires and through the Tommy’s Clinical Decision Tool itself. The NASSS framework (Non-adoption or Abandonment of technology by individuals and difficulties achieving Scale-up, Spread and Sustainability) will inform data analysis.

**Discussion:**

This paper describes the intervention, Tommy’s Clinical Decision Tool, according to TiDIER guidelines, and the protocol for the early adopter implementation evaluation study. Findings will inform future scale up.

**Trial registration:**

This study was prospectively registered on the ISRCTN registry no. 13498237, on 31^st^ January 2022.

**Supplementary Information:**

The online version contains supplementary material available at 10.1186/s12884-022-04867-w.

## Background

Poor perinatal outcomes are more common in those from ethnic minority and socially deprived groups [1]. However, even after adjustment for socio-economic and demographic characteristics, wide variation between hospital stillbirth and preterm birth rates persists [2]. This suggests that inequity in maternity care, including risk assessment and targeting of effective interventions, is an important issue that needs to be addressed. The UK’s National Health Service (NHS) Long Term Plan [3] primarily aims to ensure national programmes are focused on reducing health inequalities and addressing unwarranted variation in care, whilst empowering people to have more control over their own health, and more personalised care when they need it.

Reducing stillbirth and preterm birth rates in the UK remains a priority. In 2016 the Saving Babies’ Lives Care Bundle (SBLCB) was published by NHS England [4]. This document sets out evidence-based guidance for maternity services to achieve a UK Government target to halve stillbirths by 2030. The bundle focused on four elements: reducing smoking in pregnancy; risk assessment and surveillance for fetal growth restriction; raising awareness of reduced fetal movement and effective fetal monitoring during labour. The second version of the care bundle, published in 2019, included a new element, which focused on preventing preterm birth [5], and followed a new UK Government target to reduce the preterm birth rate from 8 to 6% by 2025 [6].

All recent national reports have identified that staff struggle with a lack of information, support and resources to provide the best care. The Each Baby Counts project investigated care related to babies who died (stillbirth or neonatal death) or suffered brain injury during birth. They found that, in 76% of cases under consideration, inadequate risk assessment and recognition was a critical contributory factor to the outcome. They also reported that in half (50%) of the cases, failure to follow guidelines or locally agreed best practice was a critical contributory factor [7]. Reasons for not following guidelines included gaps in training, lack of recognition of problems, heavy workload, staffing levels and local guidelines not being based on best available evidence.

Effective interventions can improve outcomes: aspirin can prevent placental dysfunction and preterm preeclampsia [8]; progesterone and cervical cerclage can prevent preterm birth [9, 10]. However, these interventions need to be timely, and targeted appropriately, which relies on accurate identification of women with at-risk pregnancies and equity in care provision. At present, risk assessment in pregnancy is undertaken using a checklist approach that has been used ever since the introduction of formal antenatal care. The checklist does not weigh risk factors, assess interaction between risk factors, or include risk reduction for absence of any risks. Most notably, this form of risk assessment is devoid of a personalised or numerical risk score – thereby precluding effective risk communication and personalisation of care for the individual woman.

To address the need to improve risk assessment in pregnancy and reduce inequity in service provision, the Tommy’s National Centre for Maternity Improvement developed the Tommy’s Clinical Decision Tool. The Centre is a collaboration between Tommy’s charity (Registered charity no.1060508), the Royal College of Obstetricians and Gynaecologists (RCOG), the Royal College of Midwives (RCM) and the Women’s Voices Network, with several universities (Bristol, Sheffield, St George’s London, King’s College London) and charities (Mothers for Mothers, Prompt Maternity Foundation). The Tommy’s Clinical Decision Tool is a web-based CE marked medical device that processes information directly entered by women themselves as well as clinical test results. It uses this information to more accurately, and automatically, assess a woman’s risk of preterm birth and pregnancy complications such as preeclampsia and fetal compromise which can lead to stillbirth. It does this by utilising three validated algorithms for risk assessment and clinical decision support; one for placental disorders (preeclampsia, stillbirth) [11] and two for preterm birth, one at the beginning of pregnancy [12] and the other during pregnancy if women present with symptoms of threatened preterm labour [13]. The tool not only provides individualised risk assessment at the point of care, but also recommends care pathways which are based on best practice and current national guidance. This will lead to better targeting of interventions for preventing stillbirth and preterm birth, whilst providing reassurance and avoiding unnecessary intervention for women at low risk. The Tommy’s Clinical Decision Tool provides women with access to their assessments and care recommendations, along with links to good quality, reliable information.

This programme fits with the NHS England’s Maternity Transformation Programme, which seeks to implement the vision set out in “Better Births” [14], that all pregnant women should have access to individualised and safe care, with healthcare providers harnessing advancements in digital health data management. The Tommy’s Clinical Decision Tool utilises digital technology that will ensure access to high quality care for all women, including those who are most likely to suffer the poorest outcomes.

Many promising technological innovations in health and social care are characterised by non-adoption or abandonment by individuals or by failed attempts to scale up locally, spread distantly, or sustain the innovation long term at the organisation or system level. Digital clinical decision support tools require an expanded scope of health worker engagement across the health system in order to scale them up effectively [15]. In order to address this, our implementation strategy has taken account of current evidence regarding success factors which include: a strong evidence base, professional consensus, service user and provider involvement, adequate training for clinicians, decision support results available to healthcare service users as well as providers, automatic provision of decision support and provision of decision support where and when decisions are being made [16].

**Fig. 1:**
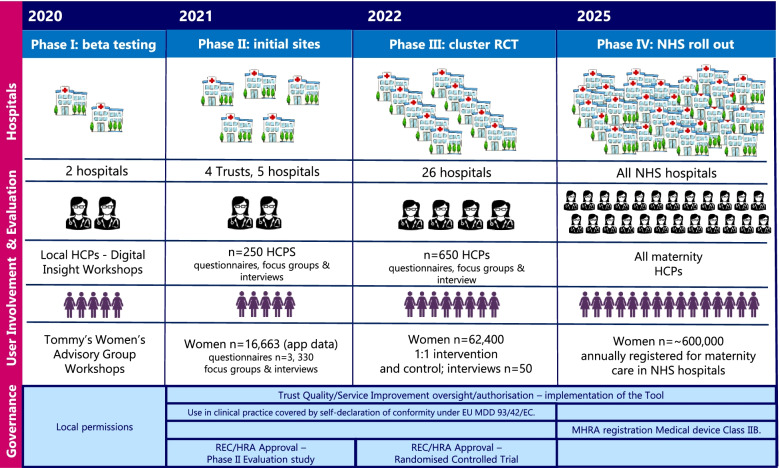
Overview of Tommy's Clinical Decision Tool Implementation and Evaluation Programme

The Tommy’s National Centre for Maternity Improvement programme aims to see implementation of the Tommy’s Clinical Decision Tool in all NHS maternity services. This will be carried out over four phases (Fig. 1). In Phase I (2020) development and beta testing of the first iteration of the tool were carried out with the Tommy’s Centre dedicated women’s involvement group, and healthcare professionals (HCPs) based in two NHS hospitals through a series of “digital workshops”. This involved initial testing and familiarisation of the prototype tool, refinements to the application and development of training packages. In Phase II, the tool is being implemented as a service development project in four early adopter NHS Trusts. In Phase III, the tool will be rolled-out to another 13 maternity services as part of a 26 site cluster randomised controlled trial (RCT), when the efficacy of the tool in the targeting of appropriate care and improved outcomes will be investigated, along with an evaluation of healthcare costs. Phase IV will see implementation of the tool in all NHS maternity services.

In this paper we provide a detailed description of the intervention, the Tommy’s Clinical Decision Tool, and describe the protocol for a mixed-methods study evaluating the implementation of the Tool in four early adopter NHS Trusts (Phase II).

## The intervention: Tommy’s Clinical Decision Tool

The Tommy’s National Centre for Maternity Improvement developed a Community of Practice (CoP) group to engage and involve relevant UK maternity care providers and users from a broad geographic and socio-demographic background in the design, development and testing of the device. The CoP formed a consensus on suitable algorithms for use as well as agreeing definitions for risk groups and corresponding personalised care pathways. They agreed that the included algorithms should ideally be taken from high quality RCTs carried out in settings relevant to the UK health care system and demonstrating consistent clinical benefit. Where such trials were unavailable, algorithms would be taken from high quality intervention studies incorporating an appropriate standard care comparison group and showing improvements in relevant clinical outcomes and/or health care resource use, establishing external validation. Two systematic reviews were conducted to identify internally and externally validated multiple marker algorithms suitable for use in risk assessment for placental dysfunction, preterm birth and threatened preterm labour [17, 18].

A systematic review of the literature identified four internally validated first trimester algorithms to predict preterm birth. All used maternal characteristics, medical and previous obstetric history. The only algorithm calculating risk of preterm birth before 34 weeks was selected [12]. The algorithm had also been externally validated in a Dutch cohort [18].

The best performing model for placental function assessment, of the 11 identified, had been developed over 15 years with data from more than 120,000 pregnancies. The regression model uses maternal characteristics (age, height, weight, ethnicity, family history of pre-eclampsia, medical and obstetric history), as well as current blood pressure, first trimester uterine artery Doppler and maternal serum PAPP-A or PlGF. The algorithm was validated in a large multi-centre NIHR trial involving over 16,000 pregnancies, and was shown to have good performance with both improved sensitivity and specificity compared to current NICE recommendations [19]. Efficacy in identifying women at high risk of pre-eclampsia, who were prescribed aspirin, with subsequent reduction of preterm pre-eclampsia, was demonstrated in a large multi-centre double-blind RCT [20]. Effectiveness in a ‘real world’ setting has also been demonstrated [11].

The algorithm used for assessment in threatened preterm labour utilises risk factors and clinical test results (fetal fibronectin and/or transvaginal ultrasound measurement of cervical length). It is recommended in the Saving Babies Lives Care Bundle v.2, and has been externally validated [13]. A prospective evaluation demonstrated that the algorithm could safely guide management and avoid hospitalisation in the vast majority (90%) of cases [21]. The current National Institute for Health and Care Excellence (NICE) guideline recommends a treat-all approach for women presenting with symptoms before 30 weeks’ gestation. If applied in this cohort, all women would have been admitted, exposing the vast majority of mothers and their babies to unnecessary hospitalisation, medical intervention and in-utero transfers.

The care pathways recommended by the Tool are taken from national guidelines, which include the Saving Babies Lives Care Bundle v.2 [4]. Table 1 column 4 summarises the care pathways that may be recommended, depending on result of the individualised risk assessment, at specific timepoints.

**Table 1:** Tommy's Clinical Decision Tool assessments, key input variables and care pathway recommendations

1. Assessment	2. Target	3. Key input variables	4. Care pathway recommendations
**Preterm birth assessment**	All women, at booking, 8–12 weeks’ gestation.	• Demographic characteristics. • NHS number. • Medical history. • Obstetric history.	• If low risk: standard care (as per NICE guidelines). • If moderate risk: cervical length at 17–21 weeks’ gestation. • If high risk: refer to preterm birth service.
**Placental function assessment**	All women at booking or before 16 weeks’ gestation.	• Demographic characteristics. • Medical history. • Blood pressure (BP). • Results from 1st trimester ultrasound scan (USS): crown rump length (CRL); uterine artery dopplers. • Blood test result: PAPP-A MoM	• If low risk: standard care (as per NICE guidelines). • If moderate risk: 2 additional USSs for fetal growth assessment and timing of birth (ToB) discussion. • If high risk: 150 mg aspirin daily, additional 3 USSs and ToB discussion.
**Changes in fetal movements (FM) assessment**	Women presenting with changes in fetal movements.	• Results of placental function assessment (auto populated). • Gestation at attendance. • Number of attendances within the last four weeks. • Presence of gestational diabetes or gestational hypertension.	*Dependent on individualised risk:* • Fetal heart auscultation (< 28 weeks). • computerised cardiotocograph (cCTG) (if had USS in last 2 weeks). • cCTG and USS. • Refer to maternal fetal medicine specialist. • Consider offering induction of labour or caesarean birth.
**Possible preterm labour assessment**	Women presenting with symptoms of threatened preterm labour.	• Demographic characteristics. • Medical history. • Obstetric history. • Fetal fibronectin (fFN) test result and/or cervical length measurement.	*Dependent on individualised risk of birth within 7 days:* • < 5%: Observation ± discharge • ≥ 5%: Steroids for fetal lung maturation and admission ± in utero transfer to hospital with available neonatal cot, if required.
**Timing of birth assessment**	Women ≥ 36 weeks identified as moderate or high risk of placental dysfunction.	• Estimated fetal weight (EFW). • Umbilical artery pulsatility index (UA PI). • Middle cerebral artery pulsatility index (MCA PI).	*Dependent on individualised risk:* • Offer birth from 40 weeks. • Offer birth from 39 weeks. • Offer birth from 37 weeks. • Refer to Specialist/Fetal Medicine.

In some circumstances, the individual risk assessment is over-ridden and the application defaults to recommend alternative pathways. These are shown in Table 2.

**Table 2:** Conditions or situations in which individual risk assessment using the Tool defaults to alternative pathways

• History of cervical surgery noted in medical history: defaults to high risk for preterm birth pathway.
• Previous baby stillborn after 24 weeks or baby born after 37 weeks weighing less than 2500g (5lb 80z) in obstetric history: defaults to high risk for placental dysfunction pathway.
• Women booking for maternity care after 13 weeks’ gestation: defaults to moderate risk for placental function pathway. This is because the placental function algorithm requires the fetal crown rump length (CRL) measurement, which is only used to date pregnancies up to 13^+^0 weeks’. After this time the fetal head circumference (HC) is used.
• Ruptured membranes on possible preterm labour assessment: defaults to high risk for preterm birth pathway.
• Gestational hypertension and/or gestational diabetes on changed fetal movements assessment. Care pathway defaults to high risk for placental dysfunction pathway.

All women are encouraged to register and to use the Tommy’s Clinical Decision Tool’s Information Hub, however, in women with multiple pregnancy, the risk assessment functions are disabled. In women with pre-existing diabetes or pre-existing hypertension, only the initial preterm birth risk assessment is available. This is because comprehensive care pathways are already established, and these women are referred to local specialist teams. Trusts may also choose to use the Tommy’s Clinical Decision Tool alongside local guidance when delivering care for women with other conditions, such as Antiphospholipid Syndrome or Chronic Kidney Disease.

**Fig. 2:**
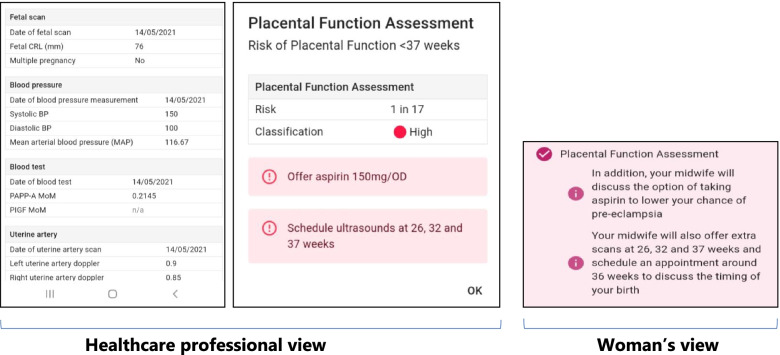
Tommy's Clinical Decision Tool dual interface: example of placental function assessment

The Tommy’s Clinical Decision Tool has a dual interface: one for use by the maternity service user, with accessible terminology, through which they can also access the Information Hub, and one for healthcare professionals, through which data is verified and/or entered by the clinician. The interfaces were designed and developed by clinicians and women, who approved use of incorporated terminology*.* Figure 2 shows an example of the dual interface following a placental function assessment.

**Fig. 3:**
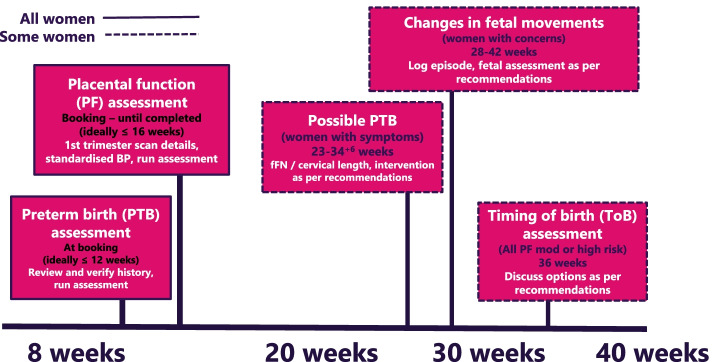
Tommy's Clinical Decision Tool touchpoints for risk assessment

Maternity service users are encouraged to register on the Tool prior to their booking appointment (i.e. the first contact with a midwife who takes a full history and makes plans for her pregnancy care). Once registered, the woman enters information about herself and her medical and obstetric history. During the appointment, the midwife checks with the woman and verifies this information, runs the preterm risk assessment, explains the recommended care pathway and makes any necessary referrals. Before the woman is 16 weeks pregnant, a healthcare professional, usually her midwife, will enter results from her first trimester ultrasound scan, blood results and blood pressure measurement (column 3, Table 1), runs the placental function assessment and actions the recommended care pathway. If the woman later experiences changes in fetal movements, or symptoms of possible preterm labour, the Tommy’s Clinical Decision Tool is used to carry out further risk assessments and generate appropriate care recommendations. An overview of touchpoints is shown in Fig. 3, while more detail on these processes is provided in Table 3.

**Table 3:**
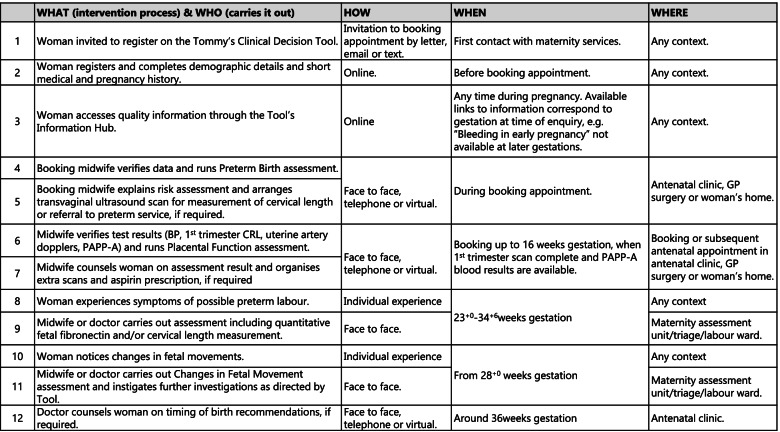
Tommy's Clinical Decision Tool—intervention processes

### Tommy’s Clinical Decision Tool Information Hub

The Information Hub provides women with links to the latest evidence-based information and guidance from trusted resources such as the NHS, Tommy’s charity and the Royal College of Obstetricians and Gynaecologists. It displays relevant links at different stages in the woman’s pregnancy, based on her gestation at the time she accesses the hub. This resource, which will be regularly reviewed and updated, currently provides links to information on the conditions and issues listed in Table 4.

**Table 4:** Conditions and issues covered in the Tommy's Clinical Decision Tool Information Hub

Information Hub Topic
Bleeding in early pregnancy
Nausea and vomiting during pregnancy
What does high and low risk mean?
Smoking in pregnancy
Alcohol in pregnancy
Healthy eating in pregnancy
Caffeine intake in pregnancy
Exercise in pregnancy
Mental wellbeing in pregnancy
Symptoms to look out for in pregnancy
High blood pressure (hypertension) and pre-eclampsia
Vaginal bleeding in pregnancy
Low-lying placenta after 20 weeks of pregnancy (placenta praevia)
Early labour (labour before 37 weeks of pregnancy)
Corticosteroids (steroids) in pregnancy
When your waters break
Changes in your baby’s movement
Gestational diabetes
Intrahepatic cholestasis of pregnancy (Itching in pregnancy)
Having a small baby (fetal growth restriction)
Planned caesarean birth
Induction of labour (also known as “induction” or “induced labour”)
Baby in the breech position
Group B Streptococcus (GBS)

### Data storage

Data collected through the Tool will initially be held by the Royal College of Obstetricians and Gynaecologists (RCOG), who will host the data in a secure, closed cloud storage in line with NHS Digital security standards. The data will then be securely migrated to NHS Digital and hosted in their Cloud Centre for Excellence (Cloud CfE).

## Methods/Design

### Study aim and objectives

#### Aim

The study is an implementation process evaluation, based on current guidance [22–24], which aims to provide evidence to support the planned ‘real world implementation’ of Tommy’s Clinical Decision Tool within the NHS.

#### Objectives

- To understand the functioning of the Tommy’s Clinical Decision Tool by specifying intervention description and implementation strategies, and examining implementation outcomes, mechanisms of impact, and contextual factors in four early adopter NHS Trusts.
- Assessment of acceptability and usability of the Tool, and acceptability of the personalised risk assessment and care recommendations it provides. This will be assessed from the perspectives of maternity service users, health professionals and organisations.
- Assessment of barriers and facilitators to successful implementation.
- Assessment of reach, i.e., whether the Tool is used by, and for risk assessment of, a representative sample of the population of maternity service users.
- Assessment of fidelity, i.e., whether the Tool is used and implementation proceeds as expected.
- Identification of unintended consequences.

### Study design

This project is a mixed-methods implementation evaluation study, which will be carried out in four NHS Trusts (five hospital sites). It consists of three Work Packages: 1. Evaluation of healthcare professionals and providers views and experience of using the Tommy’s Clinical Decision Tool; 2. Evaluation of women’s views and experience of the tool and the maternity care it has influenced and 3. Evaluation of reach and fidelity.

### Implementation outcome measures

**Fig. 4:**
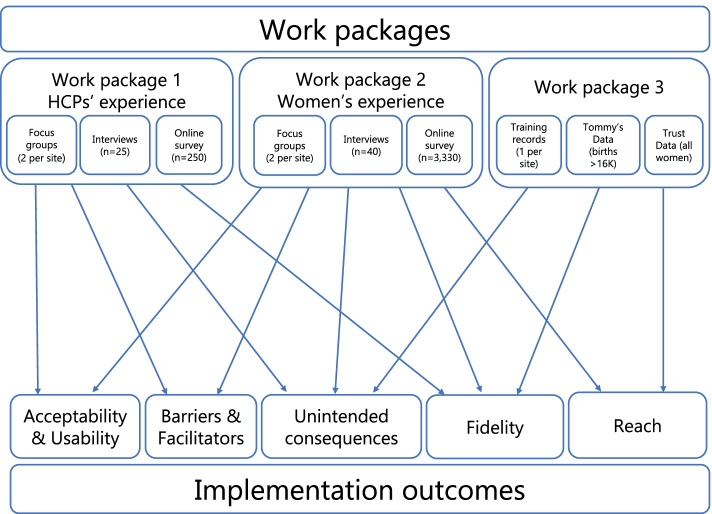
The relationship between Study Work Packages and Implementation Outcomes

1. Acceptability and usability will be measured using qualitative data from focus groups and interviews with women and healthcare professionals/providers and study specific online questionnaires developed for this study (Additional Files 1, 2 and 3), that include the mHealth App Usability Questionnaire (MAUQ) for Standalone mHealth Apps [25]. This will include evaluation of an implementation toolkit (i.e. a collection of resources to aid training and implementation).
2. Barriers and facilitators will be measured using qualitative data from focus groups and interviews and online questionnaires.
3. Reach will be measured using qualitative data from focus groups and interviews, online questionnaires and comparison of aggregate data collected through the Tool with Trust maternity data.
4. Fidelity will be measured using qualitative data from focus groups and interviews, online questionnaires, comparison of aggregate data collected through the Tool with Trust maternity data, and site records of training activity.
5. Unintended consequences will be measured using qualitative data from focus groups and interviews, online questionnaires and data collected through the Tommy’s Clinical Decision Tool and Trust maternity statistics. The relationship between the work packages and implementation outcomes is demonstrated in Fig. 4.

### Participants

#### Work Package 1 – Health professionals’ and providers’ views and experience

HCPs will be invited to complete an online questionnaire through posters/flyers and by local Champions, Research Midwives and by Trust email. Those who are willing to consider further participation in interviews or focus groups will be selected through a process of purposive sampling. This method of sampling has been chosen to ensure that the experiences of a wide range of HCPs are investigated in more detail, e.g., clinicians with little or much experience, those in management of staff or IT systems. Five HCPs from each site will be interviewed, and 5–8 HCPs will be invited to one of two focus groups per site. In total, there will be 25 HCP interviews, and 10 focus groups of 5–8 HCPs.

#### Work Package 2 – Women’s views and experience

All pregnant women choosing to book their maternity care at participating Trusts and who register to use the tool at or before their booking appointment will be eligible. Potential participants will be invited through posters/flyers and direct contact. When the women first register to use the tool they will see a page of text explaining the study. They will be asked to consider agreeing to their contact details and expected and actual date of birth of their baby being passed to researchers. All women agreeing to contact by researchers will be texted and/or emailed an invitation to complete an online questionnaire during pregnancy, and after their baby is born. At the end of the online questionnaires, participants will be given the opportunity to enter their name and email address if they are willing to consider being interviewed or participating in a focus group. To ensure a wide range of experiences and views are captured during focus groups and interviews, purposive sampling will be used to identify women with different demographic characteristics, (e.g., age group, ethnic group, parity, Index of Multiple Deprivation (IMD) decile); risk status, as determined by the Tommy’s Clinical Decision Tool (i.e., low, moderate, high); pregnancy outcome (i.e., late miscarriage or stillbirth, neonatal death, preterm birth, live birth). Based on numbers of births in participating Trusts, with a 20% response rate over 9 months data collection period, we estimate that 3,330 women will complete at least one online questionnaire. Of these, 40 women will be invited for interview and 5–8 will be invited to one of two focus groups per site. Women whose preferred language is not English will be offered the opportunity to have the interview conducted through an interpreter.

#### Work Package 3 – Evaluation of reach and fidelity

The number of records included in the aggregate data will depend on final numbers of women booking for maternity care and giving birth to their babies at the early adopter Trusts. Estimated sample size (*n* = 16,663 births) was based on average number of births at participating Trusts recorded on NHS Digital’s National Maternity Dataset.

### Data collection

#### Qualitative data

The semi-structured interviews will last around one hour and be organised at a time and place convenient to the participant. Focus groups will last around one and a half hours and be carried out either virtually (e.g. MSTeams or Zoom) or face-to-face. An interview schedule and topic guides will be used to direct the discussion on the participant’s views and experience of using the Tool, as well as their understanding of the rationale for risk assessment and recommendations for care. We will also explore how information provided through the tool was used by women to inform decisions about their care. Interviews and focus groups will be recorded on an encrypted digital recording device and/or the online platform, and uploaded to a secure server managed by a University approved transcription service. During the process of transcription, names and any information that may lead to identification of participants will be removed or changed to maintain anonymity. Transcripts will be produced and downloaded by the researchers who will store them on a secure University Microsoft Sharepoint site specific to this study.

#### Online questionnaires

 Online questionnaires will be managed through Qualtrics, a University approved online survey management system*.* The first page of the questionnaire will have brief information about the study and a link to the full participant information sheet. Participants will be asked to confirm they have read this information sheet and consent to continue. If willing, they will be asked questions about their experience of using the Tool, risk assessment, recommended care pathways and their pregnancy outcomes. They will also be asked about their use of the Tool’s Information Hub and whether it influenced any decisions they made about seeking further advice or care. The computer device identification number (IP address) will be kept in order to minimise the chance of the same participant completing a duplicate questionnaire. Other than the IP address, no identifiers will be stored, unless entered by women who consent to be contacted about the interviews or focus groups.

#### Aggregate data

Aggregate data for Work Package 3, exploring reach and fidelity, will be obtained through the Tool developer or from NHS Digital. All women booked for maternity care will be advised that their clinical data is managed according to standards approved by NHS Digital and those who do not wish their data to be used for research purposes can opt-out. This information will be made available to them via their booking letter and when they register on the Tool. Aggregate data on characteristics and outcomes of all women receiving maternity care at participating Trusts will be obtained either from individual Trusts or through NHS Digital’s publicly available Maternity Services Data Set (MSDS). The data will be used to describe and explore factors as listed in Table 5.

**Table 5:** Work Package 3: Reach and Fidelity, factors to be investigated

Factors to be investigated
All data to be reported as overall and by maternity unit—then by: 1. Age (< 16, 16–19, 20–24, 25–29, 30–34, 35–39, 40–44, 45 + years) 2. Parity (nulliparous/multiparous) 3. Ethnic Group 4. Index of Multiple Deprivation (IMD)
**Registration**
Number verified for maternity care
% of women who verified their email address but did not proceed beyond the NHS Digital “use of data” page
% of women agreed to contact from researchers
% of women who had the data they had entered corrected by the HCP
**Risk assessment (of verified women)**
% of women where preterm birth risk assessment carried out
% of women where placental function risk assessment carried out
% of women who were not eligible for risk assessment (pre-existing diabetes, pre-existing hypertension, multiple pregnancy)
% of women where changes in fetal movements assessment was carried out
% of women where recurrent changes in fetal movements assessment was carried out
% of women where threatened preterm labour assessment was carried out
% of women where timing of birth assessment was carried out
% of women assessed to be (low/moderate/high) for preterm birth
% of women assessed to be (low/moderate/high) for placental dysfunction
**Care pathway**
% of women received aspirin when indicated
% of women referred for extra ultrasound scans when indicated
% of women referred to preterm service when indicated
% of women offered induction of labour when indicated
**Information hub**
% of women who accessed at least one Information Hub page
% of women who accessed each individual page
**Pregnancy outcomes**
% of women having live birth
% of women having miscarriage
% of women having neonatal death (at time of pregnancy outcome survey completed)
% of women having stillbirth
% of women having termination of pregnancy (surgical/ medical)
% women transferring care
% of women having birth < 34wks
% of women having birth < 37wks
% of induction of labour
% emergency caesarean section
% elective caesarean section
% of women having vaginal birth
% of women having assisted birth (ventouse/ forceps)
% of women discharged while baby remained in Neonatal Unit
% of women verified who had miscarriage, stillbirth, preterm birth or neonatal death

### Data analysis

#### Qualitative data

Qualitative data collected through interviews and focus groups will be managed through NVivo qualitative data software and analysed using a thematic framework approach. The Framework approach [26] is a method of qualitative data analysis designed to generate findings that can inform practice and policy and the steps used within this approach lend themselves well to the data likely to be generated in this study. Data analysis will be informed by the NASSS (Non-adoption or Abandonment of technology by individuals and difficulties achieving Scale-up, Spread and Sustainability) framework [27], in addition to inductive analysis (where the researcher is open to new themes emerging from the data). This framework was chosen because it is an evidence-based, theory-informed and pragmatic framework to help predict and evaluate the success of a technology-supported health or social care program. A proportion of transcripts and identification of themes will be reviewed by a second experienced researcher to increase validity.

#### Online questionnaires

Statistical software will be used to explore and analyse the quantitative data gathered through the questionnaires. Participant demographic characteristics and risk profiles will be explored using descriptive statistics (i.e., frequencies and percentages) and groups will be compared using multivariate and multilevel logistic regression models. Regression models will be adjusted to account for maternal demographic and risk characteristics. Differences between groups will be considered statistically significant if the p value is ≤ 0.05. Qualitative data gathered through free text boxes, (e.g., answers to the question: “Is there anything else you would like to tell us about your experience of using the tool?”) will be analysed using qualitative thematic analysis.

#### Aggregate data

Participant characteristics, risk status, care pathways and outcomes will be explored using descriptive statistics (i.e., frequencies and percentages) and groups (i.e. those registered on the Tool and all maternity service users) will be compared using Pearson’s Chi-squared tests and multilevel logistic regression models. Regression models will be adjusted to account for maternal demographic and risk characteristics. Differences between groups will be considered statistically significant if the p value is ≤ 0.05. Apparent differences will be explored and described in detail.

### Ethical issues

#### Potential distress to participants

It is possible that some women may find recounting their experience of pregnancy and birth distressing, particularly those experiencing poor outcomes. In order to minimise any potential distress, the email texts and online questionnaires were sensitively composed and were approved by our PPI group, some of whom have experienced poor pregnancy outcomes themselves. Participants will be reminded that their participation is voluntary and they can withdraw at any time. The research team will assess whether significant risks are emerging during data collection. If they are, participants will be directed to a resources document with contact details of organisations and charities who offer support. The researcher will also offer to provide help in obtaining support from other sources, such as her GP.

#### Incentives

Healthcare professionals will be offered vouchers worth £10 for participating in interviews and £20 for participating in focus groups. Women completing the online questionnaires will be offered the opportunity to be entered into a draw for a £100 shopping voucher. Interview and focus group participants in Work Package 2 will be offered a thank-you voucher worth £20.

### Public and patient involvement

The study’s Public and Patient Involvement (PPI) Lead is a member of the Women’s Network and the Women’s Voices Involvement Panel. This group includes women with lived experience of preterm birth, stillbirth and neonatal death, women with a usual birth experience representing those not immediately considered high risk, and women with no maternity experience representing the views of first-time mothers. The group has been involved in the design and development of the Tommy’s Clinical Decision Tool and this implementation evaluation study from inception, attending workshops and Community of Practice events. They have informed the design, language, communication of risk, user experience, functionality, patient information, implementation plans as well as study participant documents and data collection instruments.

## Discussion

This study will evaluate the implementation of Phase II of the Tommy’s National Centre for Maternity Improvements’ Tommy’s Clinical Decision Tool programme. The Tool is used for risk assessment and provides evidence-based care recommendations to reduce inequalities in service provision and, ultimately, stillbirth and preterm birth. Evaluating implementation in four early adopter maternity services gives us the opportunity to evaluate implementation processes and produce findings that will inform wider scale up as, all too often, the successful implementation of a healthcare intervention in one context is not necessarily replicated in others [28].

The Tommy’s Clinical Decision Tool will allow maternity services to effectively implement the best evidenced-based care, including those pathways recommended in SBLCB v.2, with its additional element for reducing preterm birth. It will also, importantly, allow more effective targeting of these pathways through more accurate risk assessment. Widdows and colleagues [29] reported findings from a pragmatic study comparing clinical and process outcomes before and after implementation of the first version of the Saving Babies Lives Care Bundle. Although the stillbirth rate had fallen from 4.2 to 3.4 per 1,000 over the study period (aRR 0.80, (95% CI 0.70 to 0.91, *P* < 0.001), they were not able to confirm this was a direct result of the implementation of the care bundle. They did, however, report an increase in caesarean sections, inductions of labour, ultrasound scans, neonatal unit admissions and the proportion of small for gestational age infants detected. They recommended further prospective studies that could more rigorously evaluate the clinical and cost consequences of implementing SBLCB. Our evaluation of the Phase II implementation will allow us to answer some of the questions that remain unanswered by Widdows and colleagues [29], in a more rigorous prospective study. The next phase of the programme is a cluster RCT which will investigate the Tool’s effect on clinical outcomes (including rates of stillbirth and preterm birth), service outcomes (e.g. number of ultrasound scans, rates of induction and caesarean sections) and cost consequences.

A major strength of this programme is that its driving force is truly multidisciplinary in nature: a collaboration between maternity services users themselves, the professional bodies of the two main clinical disciplines in maternity care, academic expertise provided by several universities and the support of interested charities. Another strength is that the aims of the programme are in line with national policy, including the Maternity Transformation Programme [30] and the NHS Digital’s Data and information strategy [31]. Trusts implementing the Tommy’s Tool will also be able to claim a rebate from the Maternity Incentive Scheme (CNST) as the Tool is compliant with SBLCB.

We recognise that in its first iteration the Tommy’s Clinical Decision Tool is limited by a number of factors. Firstly, it is only available in the English language. Secondly, the Tool is not yet fully interactive with Trust maternity information systems. While this remains the case, healthcare professionals need to record the risk assessments and care recommendations on the Trust maternity records. These are significant barriers to successful reach and scale up, however, these issues are being addressed and resolutions will be applied to future iterations of the Tool.

The findings of this study will be reported using the Standards for Reporting Implementation Studies (StaRI) guidelines [32]. We have provided detailed description of the Tommy’s Clinical Decision Tool in this paper, but will report adaptations to the Tool, along with the implementation processes, in the main findings paper, according to the TIDieR guidelines for describing interventions [33].

## Supplementary Information

**Additional file 1:** HCP online questionnaire. Online questionnaire for completion by healthcare professionals.

**Additional file 2:** Antenatal questionnaire. Online questionnaire for completion by women participants before the birth of their baby.

**Additional file 3**: Postnatal questionnaire. Online questionnaire for completion by women participants after the birth of their baby.

## Data Availability

Not applicable. This paper describes a study protocol, and therefore contains no data.

## Abbreviations

cCTG: Computerised cardiotocograph
CFM: Changed fetal movements
CL: Cervical length
CoP: Community of Practice
CRL: Crown rump length
fFN: Fetal fibronectin
HCP: Healthcare professional/provider
KCL: King’s College London
MAUQ: MHealth App Usability Questionnaire
MoM: Multiple of the Median
NHS: National Health Service
PAPP-A: Pregnancy Associated Plasma Protein-A
PF: Placental Function
PPTL: Possible preterm labour
PTB: Preterm birth
RCT: Randomised controlled trial
SBLCB: Saving Babies Lives Care Bundle
ToB: Timing of birth
USS: Ultrasound scan
WP: Work package
