# Supplementary material for: The Tommy’s Clinical Decision Tool, a device for reducing the clinical impact of placental dysfunction and preterm birth: protocol for a mixed-methods early implementation evaluation study
Source: BMC Pregnancy Childbirth. 2022 Aug 15;22:639. doi: 10.1186/s12884-022-04867-w (PMC9377101; doi:10.1186/s12884-022-04867-w)
Supplement: Supplementary file 1 — Additional file 1: HCP online questionnaire. Online questionnaire for completion by healthcare professionals. [file 12884_2022_4867_MOESM1_ESM.pdf]

Tommy's Tool - HCP survey

Start of Block: Tommy's App - HCP survey - Pilot

Q1 The Tommy's App (Pathway) is a clinical decision tool that was designed to help doctors and midwives make sure pregnant women and people receive the right care at the right time by more accurately assessing their chance of preterm birth and placental problems which can potentially lead to pre-eclampsia, fetal growth restriction and stillbirth. You work in one of the first hospitals to use this tool. In the future, we hope that it will be available in all NHS hospitals, but before more hospitals start using it, we want to find out whether there is anything we can do to make it better. We want to hear about your views and experiences of the tool since it was launched in your hospital. You can read a copy of the information sheet here. This survey will take approximately 5 to 10 minutes. Thank you for considering taking part in this survey.

Q2 If you are happy to proceed, please confirm you have read the participant information sheet and agree to participate below:

- ○ Yes, I agree to participate. (1)
- ○ No, I do not wish to participate. (2)

End of Block: Tommy's App - HCP survey - Pilot

Start of Block: Non participation

Display This Question:

If If you are happy to proceed, please confirm you have read the participant information sheet and a... = No, I do not wish to participate.

Q3 Thank you for your time in considering this study. If you do not wish to continue, please close your browser. If you do wish to participate, please click the back button and tick the "I agree to participate" box.

End of Block: Non participation

Start of Block: About your role

Display This Question:

If If you are happy to proceed, please confirm you have read the participant information sheet and a... = Yes, I agree to participate.

Q4 Thank you for agreeing to take part in this survey. First we would like to ask some questions about you and your professional role.

Q5 In which hospital do you work?

- ◯ St Peter's Hospital, Chertsey (1)
- ◯ Royal Bolton Hospital (2)
- ◯ University Hospital Lewisham (3)
- ◯ Jessop Wing, Sheffield (4)
- ◯ Queen Elizabeth, Greenwich (5)

Page Break

Q6 What is your role?

- ◯ Midwife (1)
- ◯ Doctor (2)
- ◯ Sonographer (3)
- ◯ Other (4)

Display This Question:

If What is your role? = Other

Q7 If "Other", please state your role below.

____

Page Break

Q8 In which setting or settings do you work? (please tick all that apply)

- ☐ Community (1)
- ☐ Antenatal clinic (2)
- ☐ Ultrasound department (3)
- ☐ Maternity assessment or triage (4)
- ☐ Early pregnancy unit (5)
- ☐ Labour ward or birth centre (6)
- ☐ Antenatal ward (7)
- ☐ Postnatal ward (8)
- ☐ Caseload or Continuity team (work in all areas) (9)
- ☐ Other (12)

Page Break

Display This Question:

If In which setting or settings do you work? (please tick all that apply) = Other

Q9 If "Other", please state the setting or settings where you work below.

____

____

____

____

____

Page Break

End of Block: About your role

Start of Block: Tommy's Tool Assessments and Care Pathways

*

Q15 Please indicate below which risk assessments you have carried out using the Tommy's App (please tick all that apply).

▢ Preterm birth risk assessment (usually carried out at the booking appointment) (1)

▢ Placenta function assessment (usually carried out before 16 weeks) (2)

▢ Changes in fetal movements risk assessment (3)

▢ Possible preterm labour assessment (in women with symptoms) (4)

▢ Timing of birth assessment (for women who are at moderate or high risk of placenta dysfunction) (5)

Page Break

Display This Question:

If Please indicate below which risk assessments you have carried out using the Tommy's App (please t... = Preterm birth risk assessment (usually carried out at the booking appointment)

Q41 Please tell us about your views and experience of the preterm birth risk assessment in the box below.

____

Page Break

Display This Question:

If Please indicate below which risk assessments you have carried out using the Tommy's App (please t... = Placenta function assessment (usually carried out before 16 weeks)

Q42 Please tell us about your views and experience of the placental function risk assessment in the box below.

Page Break

Display This Question:

If Please indicate below which risk assessments you have carried out using the Tommy's App (please t... = Changes in fetal movements risk assessment

Q43 Please tell us about your views and experience of the changes in fetal movement risk assessment in the box below.

____

Page Break

Display This Question:

If Please indicate below which risk assessments you have carried out using the Tommy's App (please t... = Possible preterm labour assessment (in women with symptoms)

Q44 Please tell us about your views and experience of the possible preterm labour risk assessment in the box below.

____

____

____

____

____

Page Break

Display This Question:

If Please indicate below which risk assessments you have carried out using the Tommy's App (please t... = Timing of birth assessment (for women who are at moderate or high risk of placenta dysfunction)

Q45 Please tell us about your views and experience of the timing of birth risk assessment in the box below.

____

____

____

____

____

Page Break

Q18 Have you ever been concerned about a risk assessment result the Tommy's App provided?

- Yes (1)
- No (2)

Page Break

Display This Question:

If Have you ever been concerned about a risk assessment result the Tommy's App provided? = Yes

Q20 Please tell us a bit more about why you were concerned and what action, if any, you took, in the below text box.

____

____

____

____

____

Page Break

Q19 Have you ever been concerned about a care pathway the Tommy's App recommended?

- Yes (1)
- No (2)

Page Break

Display This Question:

If Have you ever been concerned about a care pathway the Tommy's App recommended? = Yes

Q21 Please tell us a bit more about why you were concerned and the action you took, if any, in the below text box.

____

____

____

____

____

Page Break

Q22 Have you had any problems using the Tommy's App? (technical or otherwise)

- ○ Yes (1)
- ○ No (2)

Page Break

Display This Question:

If Have you had any problems using the Tommy's App? (technical or otherwise) = Yes

Q23 Please tell us a bit more about the problems you have had using the App in the below text box.

____

____

____

____

____

End of Block: Tommy's Tool Assessments and Care Pathways

Start of Block: Training and implementation toolkit

Q24 Please indicate below which training you received about the Tommy's App (please tick all that apply)

- ▢ Face to face - in classroom (1)
- ▢ Live online (virtual) training (2)
- ▢ Online training in work time (3)
- ▢ Online training in my own time (4)
- ▢ One to one support in clinic/practice area (5)
- ▢ Other (6)
- ▢ No training (7)

Page Break

Display This Question:

If Please indicate below which training you received about the Tommy's App (please tick all that apply) = Other

Q25 If "Other", please tell us what that was in the below text box.

____

____

____

____

____

Page Break

Q26 Do you know about the Tommy's App Implementation Toolkit?

◯ No (1)

◯ Yes (2)

Page Break

Display This Question:

If Do you know about the Tommy's App Implementation Toolkit? = No

Q47 The Tommy's App Implementation Toolkit is a comprehensive collection of resources which includes training resources, videos, evidence, and quick reference guides. You can access all the resources here: [link to website] or contact your local Tommy's App (Pathway) Champion.

End of Block: Training and implementation toolkit

Start of Block: Barriers and facilitators

Q37 Do you think there is anything that could have been done differently, in your hospital, to make the implementation of the Tommy's Tool easier? If so, please tell us about this below:

____

____

____

____

____

Page Break

Q38 What do you think went well, in your hospital, regarding the implementation of the Tommy's Tool? Please tell us your thoughts below:

____

____

____

____

____

End of Block: Barriers and facilitators

Start of Block: Unexpected consequences

Q39 Is there anything that you feel could be considered an unintended consequence of implementation of the Tommy's Tool in your hospital? If so, please tell us about this in the box below.

____

____

____

____

____

End of Block: Unexpected consequences

Start of Block: Overall views about the Tool

Q1 Now we would like to know what you thought about the Tommy's App overall. Please read the below statements and indicate how much you agree or disagree with each one.

These questions are based on the mHealth App Usability Questionnaire (MAUQ) for Standalone mHealth Apps Used by Healthcare Providers- Zhou L, Bao J, Setiawan A, Saptono A, Parmanto

B, (2019), "The mHealth App Usability Questionnaire (MAUQ): Development and Validation Study", JMIR mHealth and uHealth, 7(4):e11500. DOI: 10.2196/11500. PMID: 30973342.

	N/A (1)	Strongly disagree (2)	Disagree (3)	Somewhat disagree (4)	Neither agree nor disagree (5)	Somewhat agree (6)	Agree (7)	Strongly agree (8)
The App was easy to use. (1)	○	○	○	○	○	○	○	○
It was easy for me to learn to use the App. (2)	○	○	○	○	○	○	○	○
The navigation was consistent when moving between screens. (3)	○	○	○	○	○	○	○	○
The interface of the App allowed me to use all the functions (such as entering information, viewing information) offered by the App. (4)	○	○	○	○	○	○	○	○
Whenever I made a mistake using the App, I could recover easily and quickly. (5)	○	○	○	○	○	○	○	○
I like the interface of the App. (6)	○	○	○	○	○	○	○	○

The information in the App was well organised, so I could easily find the information I needed. (7)								
The App adequately acknowledged and provided information to let me know the progress of my action. (8)								
I feel comfortable using this App in social settings. (9)								
The amount of time involved in using this App has been fitting for me. (10)								
I would use this App again. (11)								
Overall, I am satisfied with this App. (12)								
The App would be useful for my healthcare practice. (13)								

The App improved my access to delivering healthcare services. (14)	○	○	○	○	○	○	○	○
The App helped me manage my patient's health effectively. (15)	○	○	○	○	○	○	○	○
This App has all the functions and capabilities I expected it to have. (16)	○	○	○	○	○	○	○	○
I could use the App even when the Internet connection was poor or not available. (17)	○	○	○	○	○	○	○	○
This App provides an acceptable way to deliver healthcare services, such as accessing educational materials. (18)	○	○	○	○	○	○	○	○

Page Break

Q2 Is there anything else you would like to tell us about your experience of the Tommy's App and the care recommendations it provided?

____

____

____

____

____

End of Block: Overall views about the Tool

Start of Block: Prior involvement

Q10 Did you have any involvement with the Tommy's App before it was launched in your hospital? (e.g. were you involved with organising approvals, raising awareness, training, amending practice guidelines or practices before the Tool was launched).

○ Yes (1)

○ No (2)

Display This Question:

If Did you have any involvement with the Tommy's App before it was launched in your hospital? (e.g.... = Yes

Q11 If "Yes", please explain what your involvement was, below.

____

____

____

____

____

End of Block: Prior involvement

Start of Block: Final page

Q1 Thank you for taking part in this survey. The information you have provided will help us to improve the Tommy's App (Pathway) for maternity care providers in the future.
We also want to talk to a selection of healthcare professionals in more detail about their experience of the Tommy's App through interviews and focus groups.   If you would be happy for us to contact you with more details, please indicate here and enter your name and email address below. (*These details will only be used for the purpose of contacting you with more information about this study*).

Q2 I am happy to be contacted, if selected, with more information about taking part in an interview or focus group.

○ Yes, I am happy to be contacted if I am selected for interview or focus group. (1)

○ No, I would rather not be contacted about an interview or focus group. (2)

Display This Question:

If I am happy to be contacted, if selected, with more information about taking part in an interview... = Yes, I am happy to be contacted if I am selected for interview or focus group.

Q3 So we can contact you if you are selected for interview or focus group, please enter your name here:

____

Display This Question:

If I am happy to be contacted, if selected, with more information about taking part in an interview... = Yes, I am happy to be contacted if I am selected for interview or focus group.

*

Q4 ...and your email address here:

____

Page Break

End of Block: Final page
