# Supplementary material for: The Tommy’s Clinical Decision Tool, a device for reducing the clinical impact of placental dysfunction and preterm birth: protocol for a mixed-methods early implementation evaluation study
Source: BMC Pregnancy Childbirth. 2022 Aug 15;22:639. doi: 10.1186/s12884-022-04867-w (PMC9377101; doi:10.1186/s12884-022-04867-w)
Supplement: Supplementary file 2 — Additional file 2: Antenatal questionnaire. Online questionnaire for completion by women participants before the birth of their baby. [file 12884_2022_4867_MOESM2_ESM.pdf]

Tommy's App: Antenatal survey

Start of Block: Tommy's App - Antenatal survey

Q1 The Tommy's App (Pathway) is a clinical decision tool that was designed to help doctors and midwives make sure pregnant women and people receive the right care at the right time by more accurately assessing their chance of preterm birth and placental problems which can potentially lead to pre-eclampsia, fetal growth restriction and stillbirth. You have chosen to have your pregnancy care in one of the first hospitals to use this tool. In the future, we hope that it will be available in all NHS hospitals, but before more hospitals start using it, we want to find out whether there is anything we can do to make it better. When you first registered to use the App you agreed that we could email you information about the study and a link to a survey about your experience of the App and the care it recommended. You can read a copy of the information sheet here. This survey will take approximately 10 minutes. You can stop at any time. As a thank you, if you would like to be entered into our £100 voucher draw please enter your name and email address at the end of the survey.

Q2 If you are happy to proceed, please confirm you have read the participant information sheet and agree to participate below:

- ◯ Yes, I agree to participate. (1)
- ◯ No, I do not wish to participate. (2)

End of Block: Tommy's App - Antenatal survey

Start of Block: Non participation

Display This Question:

If If you are happy to proceed, please confirm you have read the participant information sheet and a... = No, I do not wish to participate.

Q3 Thank you for your time in considering this study. If you do not wish to continue, please close your browser. If you do wish to participate, please click the back button and tick the "I agree to participate" box.

Skip To: End of Survey If Thank you for your time in considering this study. If you do not wish to continue, please close y... Is Displayed

End of Block: Non participation

Start of Block: About your pregnancy

Display This Question:

If If you are happy to proceed, please confirm you have read the participant information sheet and a... = Yes, I agree to participate.

Q4 Thank you for agreeing to take part in this survey. First we would like to ask some questions about your pregnancy and your baby.

Q5 Which hospital is providing your pregnancy care (i.e. where are you booked to have your baby)?

○ St Peter's Hospital, Chertsey (1)

○ Royal Bolton Hospital (2)

○ University Hospital Lewisham (3)

○ Jessop Wing, Sheffield (4)

○ Queen Elizabeth Hospital, Greenwich (5)

Page Break

Q6 Is this your first pregnancy?

○ Yes, this is my first pregnancy. (1)

○ No, this is not my first pregnancy. (2)

Q7 How many weeks pregnant are you at the moment?

- ◯ 43 (1)
- ◯ 42 (2)
- ◯ 41 (3)
- ◯ 40 (4)
- ◯ 39 (5)
- ◯ 38 (6)
- ◯ 37 (7)
- ◯ 36 (8)
- ◯ 35 (9)
- ◯ 34 (10)
- ◯ 33 (11)
- ◯ 32 (12)
- ◯ 31 (13)
- ◯ 30 (14)
- ◯ 29 (15)
- ◯ 28 (16)
- ◯ 27 (17)
- ◯ 26 (18)
- ◯ 25 (19)
- ◯ 24 (20)
- ◯ 23 (21)

○ 22 (22)

○ 21 (23)

○ 20 (24)

○ 19 (25)

○ 18 (26)

○ 17 (27)

○ 16 (28)

○ 15 (29)

○ 14 (30)

○ 13 (31)

○ 12 (32)

Page Break

Display This Question:

If Is this your first pregnancy? = No, this is not my first pregnancy.

Q8 In any of your previous pregnancies, did you have any of the following (please tick all that apply):

We ask this because we want to understand whether previous pregnancy experiences affect how women feel about using the Tool in this pregnancy.

- ☐ Preterm birth between 35 and 37 weeks (1)
- ☐ Preterm birth at less than 34 weeks (2)
- ☐ Late miscarriage (between 14 and 23 weeks) (3)
- ☐ Pre-eclampsia (4)
- ☐ Baby didn't grow as well as expected (fetal growth restriction) (5)
- ☐ Stillbirth (baby died before birth) (6)
- ☐ Baby died shortly after birth (within the first 4 weeks of life) (7)
- ☐ Baby died within the first year of life. (8)
- ☐ All my previous pregnancies ended in miscarriage and/or terminations (9)
- ☐ None of the above problems (10)
- ☐ Other problems (please enter details on next page) (11)

Page Break

Display This Question:

If In any of your previous pregnancies, did you have any of the following (please tick all that appl... = Other problems (please enter details on next page)

Q9 Please tell us about any other problems you had in any previous pregnancies in the box below.

____

____

____

____

____

Page Break

Q10 Are you expecting more than one baby (e.g. twins, triplets) in this pregnancy?

- No, I am only expecting one baby. (1)
- Yes, I am expecting more than one baby (2)

Page Break

Q11 Before you became pregnant with this baby, did you already have diabetes or high blood pressure?

- No, I did not have diabetes or high blood pressure. (1)
- Yes, I have diabetes. (2)
- Yes, I have high blood pressure. (3)

Display This Question:

If Are you expecting more than one baby (e.g. twins, triplets) in this pregnancy? = Yes, I am expecting more than one baby

Q12 At the moment the App is not used to calculate chances of placental problems or preterm birth in women who are expecting more than one baby. This is because their care is managed according to local hospital guidelines.

These women can still use the App to access good quality information through the Information

Hub, so we would like to know if you have used this so far and, if you have, what you thought about it.

End of Block: About your pregnancy

Start of Block: About your first impressions of the App

Display This Question:

If Are you expecting more than one baby (e.g. twins, triplets) in this pregnancy? = No, I am only expecting one baby.

Q13 We would now like to ask you about your first impressions of the App. Thinking back to when you first heard about it and registered to use it: When did you first hear about the App?

- ○ Booking appointment letter. (1)
- ○ I was told about it when I attended for an appointment (2)
- ○ Saw it on a poster. (3)
- ○ Other. (4)
- ○ Don’t know/can't remember. (5)

Page Break

Display This Question:

If We would now like to ask you about your first impressions of the App. Thinking back to when you... = Other.

Q14 If "Other", please give details below.

____

____

____

____

____

Page Break

Display This Question:

If Are you expecting more than one baby (e.g. twins, triplets) in this pregnancy? = No, I am only expecting one baby.

Q15 Did you have any problems accessing or downloading it?

- ◯ No, I didn’t have any problems accessing or downloading it. (1)
- ◯ Yes, I had problems accessing or downloading it. (2)
- ◯ Don’t know/can't remember. (3)

Page Break

Display This Question:

If Did you have any problems accessing or downloading it? = Yes, I had problems accessing or downloading it.

Q16 If "Yes, I had problems accessing or downloading it.", please give details below.

____

____

____

____

____

Page Break

Display This Question:

If Are you expecting more than one baby (e.g. twins, triplets) in this pregnancy? = No, I am only expecting one baby.

Q17 Did you have any problems registering to use it (for example, with verifying your email address)?

- ◯ No, I didn’t have any problems registering to use it. (1)
- ◯ Yes, I had problems (please give details on next page) (2)
- ◯ Don’t know/can't remember (3)

Page Break

Display This Question:

If Did you have any problems registering to use it (for example, with verifying your email address)? = Yes, I had problems (please give details on next page)

Q18 If "Yes, I had problems registering to use it.", please give details below.

____

____

____

____

____

Page Break

Display This Question:

If Are you expecting more than one baby (e.g. twins, triplets) in this pregnancy? = No, I am only expecting one baby.

Q19 Did you have any problems understanding the questions it asked about previous pregnancies or medical history?

- ◯ No, I didn’t have any problems understanding the questions. (1)
- ◯ Yes, I had problems (please give details on next page) (2)
- ◯ Don’t know/can't remember (3)

Page Break

Display This Question:

If Did you have any problems understanding the questions it asked about previous pregnancies or medi... = Yes, I had problems (please give details on next page)

Q20 If "Yes, I had problems understanding the questions", please give details below.

____

____

____

____

____

Page Break

Display This Question:

If Are you expecting more than one baby (e.g. twins, triplets) in this pregnancy? = No, I am only expecting one baby.

Q21 Did you have any problems entering this information (about your previous pregnancies or medical history)?

◯ No, I didn’t have any problems entering this information. (1)

◯ Yes, I had problems (please give details on next page). (2)

◯ Don’t know/can't remember. (3)

Page Break

Display This Question:

If Did you have any problems entering this information (about your previous pregnancies or medical h... = Yes, I had problems (please give details on next page).

Q22 If "Yes, I had problems entering this information...", please give details below.

____

____

____

____

____

Page Break

Display This Question:

If Are you expecting more than one baby (e.g. twins, triplets) in this pregnancy? = No, I am only expecting one baby.

Q23 Did you feel comfortable entering this information (about your previous pregnancies or medical history)?

◯ Yes, I felt comfortable entering this information. (1)

◯ No, I didn’t feel comfortable entering this information (please give details on next page). (2)

◯ Don’t know/can't remember. (3)

◯ Would rather not say. (4)

Page Break

Display This Question:

If Did you feel comfortable entering this information (about your previous pregnancies or medical hi... = No, I didn’t feel comfortable entering this information (please give details on next page).

Q24 If "No, I didn't feel comfortable entering this information...", please give details below.

____

____

____

____

____

Page Break

Display This Question:

If Are you expecting more than one baby (e.g. twins, triplets) in this pregnancy? = No, I am only expecting one baby.

Q25 Did you have concerns about using the App?

- ○ Yes, I had concerns about using the App (please give details on next page). (1)
- ○ No, I didn’t have concerns about using the App. (2)
- ○ Don’t know/can't remember. (3)
- ○ Would rather not say. (4)

Page Break

Display This Question:

If Did you have concerns about using the App? = Yes, I had concerns about using the App (please give details on next page).

Q26 If "Yes, I had concerns about using the App...", please give details below.

____

____

____

____

____

Page Break

Display This Question:

If Are you expecting more than one baby (e.g. twins, triplets) in this pregnancy? = No, I am only expecting one baby.

Q27 Did you have any worries about the data security of the App? (for example, where and how information about you and your pregnancy was going to be stored, and/or how it would be used).

○ Yes, I had worries about the data security of the App (please give details on next page). (1)

○ No, I didn’t have worries about data security of the App. (2)

○ Don’t know/can't remember. (3)

○ Would rather not say. (4)

Page Break

Display This Question:

If Did you have any worries about the data security of the App? (for example, where and how informat... = Yes, I had worries about the data security of the App (please give details on next page).

Q28 If "Yes, I had worries about data security...", please give details below.

____

____

____

____

____

End of Block: About your first impressions of the App

Start of Block: How the App was used for assessing your chances

Display This Question:

If Are you expecting more than one baby (e.g. twins, triplets) in this pregnancy? = No, I am only expecting one baby.

Q29 We would now like to ask about the assessments carried out using the App and the care that was recommended for you (care pathway).
Thinking back to when you first saw your midwife for your booking appointment (or the first appointment when the midwife, or other healthcare professional, used the App):

Display This Question:

If Are you expecting more than one baby (e.g. twins, triplets) in this pregnancy? = No, I am only expecting one baby.

Q30 Did the midwife, or healthcare professional, go through the information you had already entered and confirm it was correct?

- ◯ Yes. (1)
- ◯ No. (2)
- ◯ Don’t know/can't remember. (3)

End of Block: How the App was used for assessing your chances

Start of Block: About preterm birth assessment

Display This Question:

If Are you expecting more than one baby (e.g. twins, triplets) in this pregnancy? = No, I am only expecting one baby.

Q31 Did the midwife, or healthcare professional, explain that the App would calculate your chances of preterm birth and that it would provide recommendations for your care?

- ◯ Yes. (1)
- ◯ No. (2)
- ◯ Don’t know/can't remember. (3)

Page Break

Display This Question:

If Are you expecting more than one baby (e.g. twins, triplets) in this pregnancy? = No, I am only expecting one baby.

Q32 For the preterm birth assessment, what care did the App recommend for you?

- ◯ Standard (routine) care. (1)
- ◯ Standard (routine) care plus ultrasound scan for measuring the length of your cervix. (2)
- ◯ Standard (routine) care plus referral to a preterm birth service. (3)
- ◯ I can't remember what care the App recommended. (4)
- ◯ I can't remember this assessment being carried out. (5)

Page Break

Display This Question:

If For the preterm birth assessment, what care did the App recommend for you? = Standard (routine) care plus ultrasound scan for measuring the length of your cervix.

Q33 Were you offered an ultrasound scan to measure the length of your cervix?

- ◯ Yes. (1)
- ◯ No. (2)
- ◯ Don’t know/can't remember. (3)

Display This Question:

If For the preterm birth assessment, what care did the App recommend for you? = Standard (routine) care plus referral to a preterm birth service.

Q34 Were you offered at least one appointment with a preterm birth service?

- ◯ Yes. (1)
- ◯ No. (2)
- ◯ Don’t know/can't remember. (3)

End of Block: About preterm birth assessment

Start of Block: About placental assessment

Display This Question:

If Are you expecting more than one baby (e.g. twins, triplets) in this pregnancy? = No, I am only expecting one baby.

And Before you became pregnant with this baby, did you already have diabetes or high blood pressure? = No, I did not have diabetes or high blood pressure.

Q35 Did the midwife, or healthcare professional, explain that the App would calculate your chances of placental problems and that it would provide recommendations for your care?

○ Yes. (1)

○ No. (2)

○ Don't know/can't remember. (3)

Page Break

Display This Question:

If Are you expecting more than one baby (e.g. twins, triplets) in this pregnancy? = No, I am only expecting one baby.

And Before you became pregnant with this baby, did you already have diabetes or high blood pressure? = No, I did not have diabetes or high blood pressure.

Q36 For the placental function assessment, what care did the App recommend for you?

○ Standard (routine) care. (1)

○ Standard (routine) care plus two extra ultrasound scans to monitor your baby's wellbeing, and an appointment around 36 weeks to discuss the timing of your baby's birth. (5)

○ Standard (routine) care plus the option of taking aspirin to lower your chance of preeclampsia, three extra ultrasound scans to monitor your baby's wellbeing, and an appointment around 36 weeks to discuss the timing of your baby's birth. (6)

○ I can't remember what care the App recommended. (7)

○ I can't remember this assessment being carried out. (8)

Page Break

Display This Question:

If For the placental function assessment, what care did the App recommend for you? = Standard (routine) care plus two extra ultrasound scans to monitor your baby's wellbeing, and an appointment around 36 weeks to discuss the timing of your baby's birth.

Q37 Were you offered two extra ultrasound scans to monitor your baby's wellbeing?

- ◯ Yes. (1)
- ◯ No. (2)
- ◯ Don't know/can't remember. (3)

Page Break

Display This Question:

If For the placental function assessment, what care did the App recommend for you? = Standard (routine) care plus two extra ultrasound scans to monitor your baby's wellbeing, and an appointment around 36 weeks to discuss the timing of your baby's birth.

Or For the placental function assessment, what care did the App recommend for you? = Standard (routine) care plus the option of taking aspirin to lower your chance of preeclampsia, three extra ultrasound scans to monitor your baby's wellbeing, and an appointment around 36 weeks to discuss the timing of your baby's birth.

Q38 Were you offered an appointment around 36 weeks to discuss the timing of your baby's birth?

- ◯ Yes. (1)
- ◯ No. (2)
- ◯ Don't know/can't remember. (3)

Page Break

Display This Question:

If For the placental function assessment, what care did the App recommend for you? = Standard (routine) care plus the option of taking aspirin to lower your chance of preeclampsia, three extra ultrasound scans to monitor your baby's wellbeing, and an appointment around 36 weeks to discuss the timing of your baby's birth.

Q39 Were you offered the option of taking aspirin to lower your chance of pre-eclampsia?

- ○ Yes. (1)
- ○ No. (2)
- ○ Don't know/can't remember. (3)

Page Break

Display This Question:

If For the placental function assessment, what care did the App recommend for you? = Standard (routine) care plus the option of taking aspirin to lower your chance of preeclampsia, three extra ultrasound scans to monitor your baby's wellbeing, and an appointment around 36 weeks to discuss the timing of your baby's birth.

Q40 Were you offered three extra ultrasound scans to monitor your baby's wellbeing?

- ○ Yes. (1)
- ○ No. (2)
- ○ Don't know/can't remember. (3)

Page Break

Display This Question:

If In any of your previous pregnancies, did you have any of the following (please tick all that appl... = Preterm birth between 35 and 37 weeks

And Before you became pregnant with this baby, did you already have diabetes or high blood pressure? = No, I did not have diabetes or high blood pressure.

Q41 Were you happy with the care pathway that the App recommended for you?

- ◯ Yes. (1)
- ◯ No. (2)
- ◯ Don’t know/can't remember. (3)

Page Break

Display This Question:

If Were you happy with the care pathway that the App recommended for you? = Yes.

Or Were you happy with the care pathway that the App recommended for you? = No.

Q42 Please tell us about why you were happy or unhappy about the care the App recommended for you.

____

____

____

____

____

End of Block: About placental assessment

Start of Block: About the Information Hub

Q43 The following questions are about your experience of using the The Information Hub (pages on the Tommy's App that direct the user to sources of good quality information).

Q44 Please indicate below which pages, if any, you looked at, at least once:

	Yes (1)	No (2)	Can't remember (3)
Bleeding in early pregnancy (1)			
Nausea and vomiting in pregnancy (5)			
What does high and low risk mean? (6)			
Smoking and pregnancy (7)			
Alcohol in pregnancy (8)			
Healthy eating for pregnancy (9)			
Exercise and pregnancy (10)			
Mental wellbeing in pregnancy (11)			
High blood pressure and pre-eclampsia			
Vaginal bleeding (13)			
Low lying placenta (14)			
Possible preterm birth (PTB) (15)			
Corticosteroids during pregnancy (16)			
Have my waters broken? (17)			
Your baby’s movements in pregnancy			
Gestational diabetes (19)			
Itching in pregnancy (Intrahepatic			
I have been told my baby is small (21)			
Planned caesarean section (22)			
Induction of labour (23)			
My baby is in the breech position (24)			
Group B Streptococcus (GBS) (25)			

Page Break

Display This Question:

If Please indicate below which pages, if any, you looked at, at least once: = Yes

Q45 If you viewed any of the information pages, please indicate below whether you agree or disagree with the following statements

	Agree (9)	Neither agree nor disagree (10)	Disagree (11)
I found the information I was looking for. (1)	○	○	○
The information I found made me feel reassured. (2)	○	○	○
The information I found made me feel anxious. (3)	○	○	○
The information I found was confusing. (4)	○	○	○
The information I found helped me to decide whether to contact my midwife for more advice. (5)	○	○	○
The information I found helped me to decide whether to go to the hospital for checks. (8)	○	○	○
The information I found helped me to raise my concerns with my midwife/doctor. (9)	○	○	○

End of Block: About the Information Hub

Start of Block: Overall views about the App

Q46 Now we would like to know what you thought about the App overall. Please read the below statements and indicate how much you agree or disagree with each one.
These questions are based on the mHealth App Usability Questionnaire (MAUQ) for Standalone mHealth Apps Used by Patients - Zhou L, Bao J, Setiawan A, Saptono A, Parmanto B, (2019), "The mHealth App Usability Questionnaire (MAUQ): Development and Validation Study", JMIR mHealth and uHealth, 7(4):e11500. DOI: 10.2196/11500. PMID: 30973342.

	N/A (1)	Strongly disagree (2)	Disagree (3)	Somewhat disagree (4)	Neither agree nor disagree (5)	Somewhat agree (6)	Agree (7)	Strongly agree (8)
The App was easy to use. (1)	○	○	○	○	○	○	○	○
It was easy for me to learn to use the App. (2)	○	○	○	○	○	○	○	○
The navigation was consistent when moving between screens. (3)	○	○	○	○	○	○	○	○
The interface of the App allowed me to use all the functions (such as entering information, viewing information) offered by the App. (4)	○	○	○	○	○	○	○	○
Whenever I made a mistake using the App, I could recover easily and quickly. (5)	○	○	○	○	○	○	○	○
I like the interface of the App. (6)	○	○	○	○	○	○	○	○
The information in the App was well organised, so I could easily find the information I needed. (7)	○	○	○	○	○	○	○	○
The App adequately acknowledged and provided information to let me know the progress of my action. (8)	○	○	○	○	○	○	○	○

I feel comfortable using this App in social settings. (9)	○	○	○	○	○	○	○	○
The amount of time involved in using this App has been fitting for me. (10)	○	○	○	○	○	○	○	○
I would use this App again. (11)	○	○	○	○	○	○	○	○
Overall, I am satisfied with this App. (12)	○	○	○	○	○	○	○	○
The App would be useful for my health and well-being. (13)	○	○	○	○	○	○	○	○
The App improved my access to healthcare services. (14)	○	○	○	○	○	○	○	○
The App helped me manage my health effectively. (15)	○	○	○	○	○	○	○	○
This App has all the functions and capabilities I expected it to have. (16)	○	○	○	○	○	○	○	○
I could use the App even when the Internet connection was poor or not available. (17)	○	○	○	○	○	○	○	○
This App provides an acceptable way to receive healthcare services, such as accessing educational materials. (18)	○	○	○	○	○	○	○	○

Page Break

Q47 Is there anything else you would like to tell us about your experience of the App and the care recommendations it made for you?

____

____

____

____

____

End of Block: Overall views about the App

Start of Block: About you

Q48 We would now like to ask you a few questions about yourself. This is so we can make sure we have heard the views of a wide range of women from different backgrounds.

Q49 Please tell us the age group you belong to.

- under 16 (1)
- 16 to 19 (2)
- 20 to 24 (3)
- 25 to 29 (4)
- 30 to 34 (5)
- 35 to 39 (6)
- 40 to 44 (7)
- 45 or over (8)
- Would rather not say (9)

Page Break

Q50 Which of the following most closely matches your Ethnicity

- Asian or Asian British (1)
- Black or Black British (2)
- Mixed (3)
- Other Ethnic Groups (4)
- White (5)
- Would rather not say (6)

Display This Question:

If Which of the following most closely matches your Ethnicity = Asian or Asian British

Q51 Asian or Asian British - Ethnic Group sub-category

- Indian (1)
- Pakistani (2)
- Bangladeshi (3)
- Any other Asian background (4)
- Would rather not say (5)

Display This Question:

If Which of the following most closely matches your Ethnicity = Black or Black British

Q52 Black or Black British - Ethnic Group sub-category

○ Caribbean (1)

○ African (2)

○ Any other Black background (3)

○ Would rather not say (4)

Display This Question:

If Which of the following most closely matches your Ethnicity = Mixed

Q53 Mixed - Ethnic Group sub-category

○ White and Black Caribbean (1)

○ White and Black African (2)

○ White and Asian (3)

○ Any other mixed background (4)

○ Would rather not say (5)

Display This Question:

If Which of the following most closely matches your Ethnicity = Other Ethnic Groups

Q54 Other - Ethnic Group sub-category

○ Chinese (1)

○ Any other ethnic group (2)

○ Would rather not say (3)

Display This Question:

If Which of the following most closely matches your Ethnicity = White

Q55 White - Ethnic Group sub-category

- ◯ British (1)
- ◯ Irish (2)
- ◯ Any other White background (3)
- ◯ Would rather not say (4)

Page Break

*

Q56 What is your postcode? (If you would prefer not to answer, please leave blank).

____

Page Break

Q57 What is your preferred language? (If you would prefer not to answer, please leave blank).

____

End of Block: About you

Start of Block: Final page

Q58 Thank you for taking part in this survey. The information you have provided will help us to improve the Tommy's App for women in the future.

We also want to talk to some women in more detail about their experience of the Tommy's App and how it may have influenced the pregnancy care they received. We will select 40 women from different backgrounds with different experiences so we can make sure we hear what a range of women feel about App. These women will be invited to talk to us through one-to-one interviews. We are also arranging group discussions (focus groups) and will invite 5-8 women to take part in one of two focus groups at each hospital.

If you would be happy for us to contact you with more details, please indicate here and enter your name and email address below. (*These details will only be used for the purpose of contacting you with more information about this study*).

Q59 I am happy to be contacted, if selected, with more information about taking part in an interview or focus group.

- ◯ Yes, I am happy to be contacted if I am selected for interview or focus group. (1)
- ◯ No, I would rather not be contacted about an interview or focus group. (2)

Display This Question:

If I am happy to be contacted, if selected, with more information about taking part in an interview... = Yes, I am happy to be contacted if I am selected for interview or focus group.

Q60 So we can contact you if you are selected for interview or focus group, please enter your name here:

____

Display This Question:

If I am happy to be contacted, if selected, with more information about taking part in an interview... = Yes, I am happy to be contacted if I am selected for interview or focus group.

*

Q61 ...and your email address here:

____

Page Break

Q62 Finally, if you would like to be entered into our thank you draw for a £100 shopping voucher, please indicate below. (*These details will only be used for the purpose of entering you into the draw*).

Q63 I would like to be entered into the £100 draw.

○ Yes, I would like to be entered into the draw. (1)

○ No, I would rather not be entered into the draw. (2)

Display This Question:

If I would like to be entered into the £100 draw. = Yes, I would like to be entered into the draw.

Q64 So we can contact you if you win the £100 draw, please enter your name here:

____

Display This Question:

If I would like to be entered into the £100 draw. = Yes, I would like to be entered into the draw.

*

Q65 and your email address here:

____

End of Block: Final page
