# Supplementary material for: The Tommy’s Clinical Decision Tool, a device for reducing the clinical impact of placental dysfunction and preterm birth: protocol for a mixed-methods early implementation evaluation study
Source: BMC Pregnancy Childbirth. 2022 Aug 15;22:639. doi: 10.1186/s12884-022-04867-w (PMC9377101; doi:10.1186/s12884-022-04867-w)
Supplement: Supplementary file 3 — Additional file 3: Postnatal questionnaire. Online questionnaire for completion by women participants after the birth of their baby. [file 12884_2022_4867_MOESM3_ESM.pdf]

Tommy's App: Postnatal survey v.3

Start of Block: Tommy's App - Postnatal survey

Q1 The Tommy’s App (Pathway) is a clinical decision tool that was designed to help doctors and midwives make sure pregnant women and people received the right care at the right time by more accurately assessing their chance of preterm birth and placental problems which can potentially lead to pre-eclampsia, fetal growth restriction and stillbirth. You chose to have your pregnancy care in one of the first hospitals to use this tool. In the future, we hope that it will be available in all NHS hospitals, but before more hospitals start using it, we want to find out whether there is anything we can do to make it better. When you first registered to use the App you agreed that we could email you information about the study and a link to a survey about your experience of the Tool and the care it recommended, after your baby was born. You can read a copy of the information sheet here. This survey will take approximately 10-15 minutes. You can stop at any time. As a thank you, if you would like to be entered into our £100 voucher draw please enter your name and email address at the end of the survey.

Q2 If you are happy to proceed, please confirm you have read the participant information sheet and agree to participate below:

- ○ Yes, I agree to participate. (1)
- ○ No, I do not wish to participate. (2)

End of Block: Tommy's App - Postnatal survey

Start of Block: Non participation

Display This Question:

If Q2 = No, I do not wish to participate.

Q3 Thank you for your time in considering this study. If you do not wish to continue, please close your browser. If you do wish to participate, please click the back button and tick the "I agree to participate" box.

Skip To: End of Survey If Q3 Is Displayed

End of Block: Non participation

Start of Block: About your pregnancy

Display This Question:

If Q2 = Yes, I agree to participate.

Q4 Thank you for agreeing to take part in this survey. First we would like to ask some questions about your pregnancy and your baby.

Q5 Which hospital provided your pregnancy care (i.e. where were you booked to have your baby)?

- ○ St Peter's Hospital, Chertsey (1)
- ○ Royal Bolton Hospital (2)
- ○ University Hospital Lewisham (3)
- ○ Jessop Wing, Sheffield (4)
- ○ Queen Elizabeth Hospital, Greenwich (5)

Q6 Was this your first pregnancy?

- ○ Yes, this was my first pregnancy. (1)
- ○ No, this was not my first pregnancy. (2)

Display This Question:

If Q6 = No, this was not my first pregnancy.

Q7 In any of your previous pregnancies, did you have any of the following (please tick all that apply):
We ask this because we want to understand whether previous pregnancy experiences affect how women feel about using the Tool in this pregnancy.

- ▢ Preterm birth between 35 and 37 weeks (1)
- ▢ Preterm birth at less than 34 weeks (2)
- ▢ Late miscarriage (between 14 and 23 weeks) (3)
- ▢ Pre-eclampsia (4)
- ▢ Baby didn't grow as well as expected (fetal growth restriction) (5)
- ▢ Stillbirth (baby died before birth) (6)
- ▢ Baby died shortly after birth (within the first 4 weeks of life) (7)
- ▢ Baby died within the first year of life. (8)
- ▢ All my previous pregnancies ended in miscarriage and/or terminations (9)
- ▢ None of the above problems (10)
- ▢ Other problems (please enter details on next page) (11)

Page Break

Display This Question:

If Q7 = Other problems (please enter details on next page)

Q8 Please tell us about any other problems you had in any previous pregnancies in the box below.

____

____

____

____

____

Q9 Were you expecting more than one baby (e.g. twins, triplets) in this pregnancy (the one when you used the Tommy's App).

○ No, I was only expecting one baby. (1)

○ Yes, I was expecting more than one baby (2)

Display This Question:

If Q9 = No, I was only expecting one baby.

Q10 Before you became pregnant with this baby, did you already have diabetes or high blood pressure?

▢ No, I did not have diabetes or high blood pressure. (1)

▢ Yes, I have diabetes. (2)

▢ Yes, I have high blood pressure. (3)

Page Break

Display This Question:

If Q9 = Yes, I was expecting more than one baby

Q11 At the moment the App is not used to calculate chances of placental problems or preterm birth in women who are expecting more than one baby. This is because their care would have been managed according to local hospital guidelines.

These women could still use the App to access good quality information through the Information Hub, so we would like to know if you used this and, if you did, what you thought about it.

End of Block: About your pregnancy

Start of Block: About this baby

Display This Question:

If Q9 = No, I was only expecting one baby.

And Q10 = No, I did not have diabetes or high blood pressure.

Or Q10 = Yes, I have diabetes.

Or Q10 = Yes, I have high blood pressure.

Q12 In which month was your baby born?

- ○ August 2021 (1)
- ○ September 2021 (2)
- ○ October 2021 (3)
- ○ November 2021 (4)
- ○ December 2021 (5)
- ○ January 2022 (6)
- ○ February 2022 (7)
- ○ March 2022 (8)
- ○ April 2022 (9)
- ○ May 2022 (10)
- ○ June 2022 (11)
- ○ July 2022 (12)
- ○ August 2022 (13)
- ○ September 2022 (14)
- ○ October 2022 (16)
- ○ November 2022 (17)
- ○ December 2022 (18)
- ○ January 2023 (19)
- ○ February (20)
- ○ March 2023 (21)
- ○ April 2023 (22)

○ May 2023 (23)

Display This Question:

If Q9 = No, I was only expecting one baby.

And Q10 = No, I did not have diabetes or high blood pressure.

Or Q10 = Yes, I have diabetes.

Or Q10 = Yes, I have high blood pressure.

Q13 How many weeks pregnant were you when you baby was born?

- 44 (1)
- 43 (2)
- 42 (3)
- 41 (4)
- 40 (5)
- 39 (6)
- 38 (7)
- 37 (8)
- 36 (9)
- 35 (10)
- 34 (11)
- 33 (12)
- 32 (13)
- 31 (14)
- 30 (15)
- 29 (16)
- 28 (17)
- 27 (18)
- 26 (19)
- 25 (20)
- 24 (21)

- ○ 23 (22)
- ○ 22 (23)
- ○ 21 (24)
- ○ 20 (25)
- ○ 19 (26)
- ○ 18 (27)
- ○ 17 (28)
- ○ 16 (29)
- ○ 15 (30)
- ○ 14 (31)
- ○ 13 (32)
- ○ 12 (33)
- ○ 11 (34)
- ○ 10 (35)
- ○ 9 (36)

Page Break

Display This Question:

If Q9 = No, I was only expecting one baby.

And Q10 = No, I did not have diabetes or high blood pressure.

Or Q10 = Yes, I have diabetes.

Or Q10 = Yes, I have high blood pressure.

Q14 *Please select the statement below that is most true about your baby:*

- ○ My baby is well and came home with me (or stayed with me following homebirth). (1)
- ○ My baby spent some time in the neonatal unit but came home with me. (2)
- ○ My baby is still in the neonatal unit. (3)
- ○ My baby died before birth. (4)
- ○ My baby died after they were born. (5)
- ○ Other (6)

Display This Question:

If Q14 = Other

Q15 If "Other", please give details below.

____

____

____

____

____

Page Break

Display This Question:

If Q14 = My baby died before birth.

Or Q14 = My baby died after they were born.

Q16 We are very sorry to hear that your baby died, and we would like to thank you very much for taking the time to answer these questions. It is very important that we can understand the experience of all women who received pregnancy care influenced by the App and this includes women whose babies have died as well as those who have not. However, please remember you are free to stop at any time, there is no need to complete the survey. If you would like more support, you can find some information and contact details about organisations that might be able to help you here.

Page Break

Display This Question:

If Q9 = No, I was only expecting one baby.

And Q10 = No, I did not have diabetes or high blood pressure.

Q17 How did your labour start?

- ○ Spontaneously (on its own). (1)
- ○ Induction (started by doctor/midwife). (2)
- ○ No labour, I had a caesarean section before labour. (3)
- ○ Don’t know/can't remember. (4)
- ○ Would rather not say. (5)
- ○ Other. (6)

Page Break

Display This Question:

If Q17 = Other.

Q18 If "Other", please give details below.

____

____

____

____

____

Page Break

Display This Question:

If Q9 = No, I was only expecting one baby.

And Q10 = No, I did not have diabetes or high blood pressure.

Q19 Where was your baby born?

- In hospital – labour ward/birth centre (1)
- In hospital – midwifery led/low risk birth centre. (2)
- In hospital – not sure what type of unit. (3)
- In a midwife-led unit not attached to the hospital. (4)
- At home. (5)
- Somewhere else (please give details on next page) (6)
- Don’t know/can't remember. (7)
- Would rather not say. (8)

Page Break

Display This Question:

If Q19 = Somewhere else (please give details on next page)

Q20 If "Somewhere else", please give details below.

____

____

____

____

____

Page Break

Display This Question:

If Q9 = No, I was only expecting one baby.

And Q10 = No, I did not have diabetes or high blood pressure.

Q21 How was your baby born?

- ○ Unassisted vaginal birth. (1)
- ○ Forceps or ventouse (suction). (2)
- ○ Caesarean section before labour. (3)
- ○ Caesarean section in labour. (4)
- ○ Other. (5)
- ○ Don’t know/can't remember. (6)
- ○ Would rather not say. (7)

Page Break

Display This Question:

If Q21 = Other.

Q22 If "Other", please give details below.

____

____

____

____

____

End of Block: About this baby

Start of Block: Previous survey participation

Display This Question:

If Q9 = No, I was only expecting one baby.

Or Q10 = No, I did not have diabetes or high blood pressure.

Q23 Did you complete the Tommy's App Antenatal survey? (if you tick yes below, you will not have to answer the same questions that we asked about your views and experiences of the initial preterm birth and placental function assessments and care they recommended).

- ○ No, I did not complete the Tommy's App Antenatal online survey. (1)
- ○ Yes, I did complete the Tommy's App Antenatal online survey. (2)

If “Yes...” skip to Qs on PTB and PF assessment “Did you have [care as recommended]...” questions and continue to Block: Unscheduled visits.

End of Block: Previous survey participation

Start of Block: About your first impressions of the App

Display This Question:

If Q9 = No, I was only expecting one baby.

Q24 We would now like to ask you about your first impressions of the App. Thinking back to when you first heard about it and registered to use it: When did you first hear about the App?

- ○ Booking appointment letter. (1)
- ○ I was told about it when I attended for an appointment. (2)
- ○ Saw it on a poster. (3)
- ○ Other. (4)
- ○ Don’t know/can't remember. (5)

Page Break

Display This Question:

If Q24 = Other.

Q25 If "Other", please give details below.

____

____

____

____

____

Page Break

Display This Question:

If Q9 = No, I was only expecting one baby.

Q26 Did you have any problems accessing or downloading it?

- ○ No, I didn’t have any problems accessing or downloading it. (1)
- ○ Yes, I had problems accessing or downloading it. (2)
- ○ Don’t know/can't remember. (3)

Page Break

Display This Question:

If Q26 = Yes, I had problems accessing or downloading it.

Q27 If "Yes, I had problems accessing or downloading it.", please give details below.

____

____

____

Page Break

Display This Question:

If Q9 = No, I was only expecting one baby.

Q28 Did you have any problems registering to use it (for example, with verifying your email address)?

- ◯ No, I didn’t have any problems registering to use it. (1)
- ◯ Yes, I had problems (please give details on next page) (2)
- ◯ Don’t know/can't remember (3)

Page Break

Display This Question:

If Q28 = Yes, I had problems (please give details on next page)

Q29 If "Yes, I had problems registering to use it.", please give details below.

____

____

____

____

____

Page Break

Display This Question:

If Q9 = No, I was only expecting one baby.

Q30 Did you have any problems understanding the questions it asked about previous pregnancies or medical history?

- ○ No, I didn’t have any problems understanding the questions. (1)
- ○ Yes, I had problems (please give details on next page) (2)
- ○ Don’t know/can't remember (3)

Page Break

Display This Question:

If Q30 = Yes, I had problems (please give details on next page)

Q31 If "Yes, I had problems understanding the questions", please give details below.

____

____

____

____

____

Page Break

Display This Question:

If Q9 = No, I was only expecting one baby.

Q32 Did you have any problems entering this information (about your previous pregnancies or medical history)?

- ○ No, I didn’t have any problems entering this information. (1)
- ○ Yes, I had problems (please give details on next page). (2)
- ○ Don’t know/can't remember. (3)

Page Break

Display This Question:

If Q32 = Yes, I had problems (please give details on next page).

Q33 If "Yes, I had problems entering this information...", please give details below.

____

____

____

____

____

Page Break

Display This Question:

If Q9 = No, I was only expecting one baby.

Q34 Did you feel comfortable entering this information (about your previous pregnancies or medical history)?

- ◯ Yes, I felt comfortable entering this information. (1)
- ◯ No, I didn't feel comfortable entering this information (please give details on next page). (2)
- ◯ Don't know/can't remember. (3)
- ◯ Would rather not say. (4)

Page Break

Display This Question:

If Q34 = No, I didn't feel comfortable entering this information (please give details on next page).

Q35 If "No, I didn't feel comfortable entering this information...", please give details below.

____

____

____

____

____

Page Break

Display This Question:

If Q9 = No, I was only expecting one baby.

Q36 Did you have concerns about using the App?

- ○ Yes, I had concerns about using the App (please give details on next page). (1)
- ○ No, I didn’t have concerns about using the App. (2)
- ○ Don’t know/can't remember. (3)
- ○ Would rather not say. (4)

Page Break

Display This Question:

If Q36 = Yes, I had concerns about using the App (please give details on next page).

Q37 If "Yes, I had concerns about using the App...", please give details below.

____

____

____

____

____

Page Break

Display This Question:

If Q9 = No, I was only expecting one baby.

Q38 Did you have any worries about the data security of the App? (for example, where and how information about you and your pregnancy was going to be stored, and/or how it would be used).

- ◯ Yes, I had worries about the data security of the App (please give details on next page). (1)
- ◯ No, I didn’t have worries about data security of the App. (2)
- ◯ Don’t know/can't remember. (3)
- ◯ Would rather not say. (4)

Page Break

Display This Question:

If Q38 = Yes, I had worries about the data security of the App (please give details on next page).

Q39 If "Yes, I had worries about data security...", please give details below.

____

____

____

____

____

End of Block: About your first impressions of the App

Start of Block: How the App was used for assessing your chances

Display This Question:

If Q9 = No, I was only expecting one baby.

Q40 We would now like to ask about the assessments carried out using the App and the care that was recommended for you (care pathway).
Thinking back to when you first saw your midwife for your booking appointment (or the first appointment when the midwife, or other healthcare professional, used the App):

Display This Question:

If Q9 = No, I was only expecting one baby.

Q41 Did the midwife, or healthcare professional, go through the information you had already entered and confirm it was correct?

- Yes. (1)
- No. (2)
- Don’t know/can't remember. (3)

End of Block: How the App was used for assessing your chances

Start of Block: About preterm birth assessment

Display This Question:

If Q9 = No, I was only expecting one baby.

Q42 Did the midwife, or healthcare professional, explain that the App would calculate your chances of preterm birth and that it would provide recommendations for your care?

- Yes. (1)
- No. (2)
- Don’t know/can't remember. (3)

Page Break

Display This Question:

If Q9 = No, I was only expecting one baby.

Q43 For the preterm birth assessment, what care did the App recommend for you?

- ○ Standard (routine) care. (1)
- ○ Standard (routine) care plus ultrasound scan for measuring the length of your cervix. (2)
- ○ Standard (routine) care plus referral to a preterm birth service. (3)
- ○ I can't remember what care the App recommended. (4)
- ○ I can't remember this assessment being carried out. (5)

Page Break

Display This Question:

If Q43 = Standard (routine) care plus ultrasound scan for measuring the length of your cervix.

Q44 Did you have an ultrasound scan to measure the length of your cervix?

- ○ Yes. (1)
- ○ No. (2)
- ○ Don’t know/can't remember. (3)

Display This Question:

If Q43 = Standard (routine) care plus referral to a preterm birth service.

Q45 Did you have at least one appointment with a preterm birth service?

- ○ Yes. (1)
- ○ No. (2)
- ○ Don’t know/can't remember. (3)

End of Block: About preterm birth assessment

Start of Block: About placental assessment

Display This Question:

If Q9 = No, I was only expecting one baby.

And Q10 = No, I did not have diabetes or high blood pressure.

Q46 Did the midwife, or healthcare professional, explain that the App would calculate your chances of placental problems and that it would provide recommendations for your care?

- Yes. (1)
- No. (2)
- Don’t know/can't remember. (3)

Page Break

Display This Question:

If Q9 = No, I was only expecting one baby.

And Q10 = No, I did not have diabetes or high blood pressure.

Q47 For the placental function assessment, what care did the App recommend for you?

- Standard (routine) care. (1)
- Standard (routine) care plus two extra ultrasound scans to monitor your baby’s wellbeing, and an appointment around 36 weeks to discuss the timing of your baby’s birth. (5)
- Standard (routine) care plus the option of taking aspirin to lower your chance of preeclampsia, three extra ultrasound scans to monitor your baby’s wellbeing, and an appointment around 36 weeks to discuss the timing of your baby’s birth. (6)
- I can't remember what care the App recommended. (7)
- I can't remember this assessment being carried out. (8)

Page Break

Display This Question:

If Q47 = Standard (routine) care plus two extra ultrasound scans to monitor your baby’s wellbeing, and an appointment around 36 weeks to discuss the timing of your baby’s birth.

Q48 Did you have two extra ultrasound scans to monitor your baby’s wellbeing?

- ◯ Yes. (1)
- ◯ No. (2)
- ◯ Don’t know/can't remember. (3)

Page Break

Display This Question:

If Q47 = Standard (routine) care plus two extra ultrasound scans to monitor your baby’s wellbeing, and an appointment around 36 weeks to discuss the timing of your baby’s birth.

Or Q47 = Standard (routine) care plus the option of taking aspirin to lower your chance of preeclampsia, three extra ultrasound scans to monitor your baby’s wellbeing, and an appointment around 36 weeks to discuss the timing of your baby’s birth.

Q49 Did you have an appointment around 36 weeks to discuss the timing of your baby’s birth?

- ◯ Yes. (1)
- ◯ No. (2)
- ◯ Don’t know/can't remember. (3)

Q50 How did you feel about having this discussion? Please tell us your thoughts about it in the box below.

____

____

____

____

____

Page Break

Display This Question:

If Q47 = Standard (routine) care plus the option of taking aspirin to lower your chance of preeclampsia, three extra ultrasound scans to monitor your baby's wellbeing, and an appointment around 36 weeks to discuss the timing of your baby's birth.

Q51 Were you offered the option of taking aspirin to lower your chance of pre-eclampsia?

- ◯ Yes. (1)
- ◯ No. (2)
- ◯ Don't know/can't remember. (3)

Page Break

Display This Question:

If Q47 = Standard (routine) care plus the option of taking aspirin to lower your chance of preeclampsia, three extra ultrasound scans to monitor your baby's wellbeing, and an appointment around 36 weeks to discuss the timing of your baby's birth.

Q52 Did you have these three extra ultrasound scans to monitor your baby's wellbeing?

- ◯ Yes. (1)
- ◯ No. (2)
- ◯ Don't know/can't remember. (3)

Page Break

Display This Question:

If Q9 = No, I was only expecting one baby.

And Q10 = No, I did not have diabetes or high blood pressure.

Q53 Were you happy with the care pathway that the App recommended for you?

- ○ Yes. (1)
- ○ No. (2)
- ○ Don’t know/can't remember. (3)

Page Break

Display This Question:

If Q53 = Yes.

Or Q53 = No.

Q54 Please tell us about why you were happy or unhappy about the care the App recommended for you.

____

____

____

____

____

End of Block: About placental assessment

Start of Block: About PTB assmt for women who have completed the ANQ [*in the ANQ, the participant is asked about their experience of these earlier risk assessments and the care than was planned – the following questions ask if they actually had the care that was planned (which they wouldn’t have been able to answer on the ANQ). Women who did not complete the ANQ will not see this block, or the next block “About PF assmt and ToB for women who have completed the ANQ”..*

Page Break

Display This Question:

If Q9 = No, I was only expecting one baby.

And Q23 = Yes, I did complete the Tommy's App Antenatal online survey.

Q55 For the preterm birth assessment carried out between 8 and 20 weeks of pregnancy, what care did the App recommend for you?

○ Standard (routine) care. (1)

○ Standard (routine) care plus ultrasound scan for measuring the length of your cervix. (2)

○ Standard (routine) care plus referral to a preterm birth service. (3)

○ I can't remember what care the App recommended. (4)

○ I can't remember this assessment being carried out. (5)

Page Break

Display This Question:

If Q55 = Standard (routine) care plus ultrasound scan for measuring the length of your cervix.

Q56 Did you have an ultrasound scan to measure the length of your cervix?

○ Yes. (1)

○ No. (2)

○ Don’t know/can't remember. (3)

Display This Question:

If Q55 = Standard (routine) care plus referral to a preterm birth service.

Q57 Did you have at least one appointment with a preterm birth service?

- Yes. (1)
- No. (2)
- Don’t know/can't remember. (3)

End of Block: About PTB assmt for women who have completed the ANQ

Start of Block: About PF USS & ToB for women who have completed ANQ

Page Break

Display This Question:

If Q9 = No, I was only expecting one baby.

And Q10 = No, I did not have diabetes or high blood pressure.

And Q23 = Yes, I did complete the Tommy's App Antenatal online survey.

Q58 For the placental function assessment, which was carried out before you were 20 weeks pregnant, what care did the App recommend for you?

- Standard (routine) care. (1)
- Standard (routine) care plus two extra ultrasound scans to monitor your baby’s wellbeing, and an appointment around 36 weeks to discuss the timing of your baby’s birth. (5)
- Standard (routine) care plus the option of taking aspirin to lower your chance of preeclampsia, three extra ultrasound scans to monitor your baby’s wellbeing, and an appointment around 36 weeks to discuss the timing of your baby’s birth. (6)
- I can't remember what care the App recommended. (7)
- I can't remember this assessment being carried out. (8)

Page Break

Display This Question:

If Q58 = Standard (routine) care plus two extra ultrasound scans to monitor your baby's wellbeing, and an appointment around 36 weeks to discuss the timing of your baby's birth.

Q59 Did you have two extra ultrasound scans to monitor your baby's wellbeing?

- ◯ Yes. (1)
- ◯ No. (2)
- ◯ Don't know/can't remember. (3)

Page Break

Display This Question:

If Q58 = Standard (routine) care plus the option of taking aspirin to lower your chance of preeclampsia, three extra ultrasound scans to monitor your baby's wellbeing, and an appointment around 36 weeks to discuss the timing of your baby's birth.

Q60 Were you offered the option of taking aspirin to lower your chance of pre-eclampsia?

- ◯ Yes. (1)
- ◯ No. (2)
- ◯ Don't know/can't remember. (3)

Display This Question:

If Q58 = Standard (routine) care plus the option of taking aspirin to lower your chance of preeclampsia, three extra ultrasound scans to monitor your baby's wellbeing, and an appointment around 36 weeks to discuss the timing of your baby's birth.

Q61 Did you have these three extra ultrasound scans to monitor your baby's wellbeing?

- ◯ Yes. (1)
- ◯ No. (2)
- ◯ Don't know/can't remember. (3)

Page Break

Display This Question:

If Q58 = Standard (routine) care plus two extra ultrasound scans to monitor your baby's wellbeing, and an appointment around 36 weeks to discuss the timing of your baby's birth.

Or Q58 = Standard (routine) care plus the option of taking aspirin to lower your chance of preeclampsia, three extra ultrasound scans to monitor your baby's wellbeing, and an appointment around 36 weeks to discuss the timing of your baby's birth.

Q62 Did you have an appointment around 36 weeks to discuss the timing of your baby's birth?

- ◯ Yes. (1)
- ◯ No. (2)
- ◯ Don't know/can't remember. (3)

Display This Question:

If Q62 = Yes.

Q63 How did you feel about having this discussion? Please tell us your thoughts about it in the box below.

____

____

____

____

____

Page Break

End of Block: About PF USS & ToB for women who have completed ANQ

Start of Block: Unscheduled visits TPTL

Display This Question:

If Q9 = No, I was only expecting one baby.

And Q10 = No, I did not have diabetes or high blood pressure.

Q64 The App can be used to provide assessments later on in pregnancy if a woman has symptoms that may be preterm labour (e.g. pain and/or tightenings before 37 weeks).

Display This Question:

If Q9 = No, I was only expecting one baby.

And Q10 = No, I did not have diabetes or high blood pressure.

Q65 Please indicate below whether you had any symptoms that may have been preterm labour (before 37 weeks of pregnancy)?

- ○ Yes, I had symptoms that may have been preterm labour. (1)
- ○ No, I did not have symptoms that may have been preterm labour. (2)

Page Break

Display This Question:

If Q65 = Yes, I had symptoms that may have been preterm labour.

Q66 When you had symptoms that may have been preterm labour, did you do any of the following (you can tick as many boxes as you want to):

- ☐ Looked up information about it on the Tommy's App Information Hub. (1)
- ☐ Looked up information on other websites. (2)
- ☐ Talked about it with friends and/or family. (3)
- ☐ Spoke to your midwife/doctor about it. (4)
- ☐ Don’t know/can’t remember. (5)
- ☐ Would rather not say. (6)

Page Break

Display This Question:

If Q65 = Yes, I had symptoms that may have been preterm labour.

Q67 Did you go to the hospital for further checks?

- ○ Yes (1)
- ○ No (2)

Display This Question:

If Q67 = Yes

Q68 If yes, what tests were carried out? (please enter details below)

____

____

____

____

____

Page Break

Display This Question:

If Q67 = Yes

Q69 Did the healthcare professional looking after you (e.g. midwife, doctor, sonographer) use the App to help them to decide what care to offer you?

○ Yes (1)

○ No (2)

○ Don't know/can't remember. (3)

Page Break

Display This Question:

If Q67 = Yes

Q70 What happened after that (e.g. did you stay in hospital, were you sent home? (please enter details below)

____

____

____

____

____

Page Break

Display This Question:

If Q65 = Yes, I had symptoms that may have been preterm labour.

Q71 Can you tell us a bit more about why you did or didn't go for further checks? (please enter details below)

____

____

____

____

____

End of Block: Unscheduled visits TPTL

Start of Block: Unscheduled visits CFM

Display This Question:

If Q9 = No, I was only expecting one baby.

And Q10 = No, I did not have diabetes or high blood pressure.

Q72 The App can also be used to provide assessments if a woman notices changes in her baby's movements during her pregnancy.

Display This Question:

If Q9 = No, I was only expecting one baby.

And Q10 = No, I did not have diabetes or high blood pressure.

Q73 Please indicate below whether you noticed changes in your baby's movements during pregnancy?

- ◯ Yes, I noticed changes in my baby's movements. (1)
- ◯ No, I did not notice any changes in my baby's movements. (2)

Page Break

Display This Question:

If Q73 = Yes, I noticed changes in my baby's movements.

Q74 When you noticed these changes in your baby's movements, did you do any of the following (you can tick as many boxes as you want to):

- ▢ Looked up information about it on the Tommy’s App Information Hub. (1)
- ▢ Looked up information on other websites. (2)
- ▢ Talked about it with friends and/or family. (3)
- ▢ Spoke to your midwife/doctor about it. (4)
- ▢ Don’t know/can’t remember. (5)
- ▢ Would rather not say. (6)

Page Break

Display This Question:

If Q73 = Yes, I noticed changes in my baby's movements.

Q75 Did you go to the hospital for further checks?

- ○ Yes (1)
- ○ No (2)

Display This Question:

If Q75 = Yes

Q76 If yes, what tests were carried out? (please enter details below)

____

____

____

____

____

Page Break

Display This Question:

If Q75 = Yes

Q77 Did the healthcare professional looking after you (e.g. midwife, doctor, sonographer) use the App to help them to decide what care to offer you?

- Yes (1)
- No (2)
- Don't know/can't remember. (3)

Page Break

Display This Question:

If Q75 = Yes

Q78 What happened after that (e.g. did you stay in hospital, were you sent home? (please enter details below)

____

____

____

____

____

Page Break

Display This Question:

If Q73 = Yes, I noticed changes in my baby's movements.

Q79 Can you tell us a bit more about why you did or didn't go for further checks? (please enter details below)

____

____

____

____

____

End of Block: Unscheduled visits CFM

Start of Block: About the Information Hub

Q80 The following questions are about your experience of using the The Information Hub (pages on the App that direct the user to sources of good quality information).

Q81 Please indicate below which pages, if any, you looked at, at least once:

	Yes (1)	No (2)	Can't remember (3)
Bleeding in early pregnancy (1)			
Nausea and vomiting in pregnancy (5)			
What does high and low risk mean? (6)			
Smoking and pregnancy (7)			
Alcohol in pregnancy (8)			
Healthy eating for pregnancy (9)			
Exercise and pregnancy (10)			
Mental wellbeing in pregnancy (11)			
High blood pressure and pre-eclampsia (12)			
Vaginal bleeding (13)			
Low lying placenta (14)			
Possible preterm birth (PTB) (15)			
Corticosteroids during pregnancy (16)			
Have my waters broken? (17)			
Your baby's movements in pregnancy (18)			
Gestational diabetes (19)			
Itching in pregnancy (Intrahepatic			
I have been told my baby is small (21)			
Planned caesarean section (22)			
Induction of labour (23)			
My baby is in the breech position (24)			
Group B Streptococcus (GBS) (25)			

Display This Question:

If Q81 = Yes

Q82 If you viewed any of the information pages, please indicate below whether you agree or disagree with the following statements

	Agree (9)	Neither agree nor disagree (10)	Disagree (11)
I found the information I was looking for. (1)	○	○	○
The information I found made me feel reassured. (2)	○	○	○
The information I found made me feel anxious. (3)	○	○	○
The information I found was confusing. (4)	○	○	○
The information I found helped me to decide whether to contact my midwife for more advice. (5)	○	○	○
The information I found helped me to decide whether to go to the hospital for checks. (8)	○	○	○
The information I found helped me to raise my concerns with my midwife/doctor. (9)	○	○	○

End of Block: About the Information Hub

Start of Block: Overall views about the Tool

Q83 Now we would like to know what you thought about the Tommy's App overall. Please read the below statements and indicate how much you agree or disagree with each one.

These questions are based on the mHealth App Usability Questionnaire (MAUQ) for Standalone mHealth Apps Used by Patients - Zhou L, Bao J, Setiawan A, Saptono A, Parmanto B, (2019), "The mHealth App Usability Questionnaire (MAUQ): Development and

Validation Study", JMIR mHealth and uHealth, 7(4):e11500. DOI: 10.2196/11500. PMID: 30973342. Usability Questionnaire (MAUQ): Development and Validation Study", JMIR mHealth and uHealth, 7(4):e11500. DOI: 10.2196/11500. PMID: 30973342.

	N/A (1)	Strongly disagree (2)	Disagree (3)	Somewhat disagree (4)	Neither agree nor disagree (5)	Somewhat agree (6)	Agree (7)	Strongly agree (8)
The App was easy to use. (1)	○	○	○	○	○	○	○	○
It was easy for me to learn to use the App. (2)	○	○	○	○	○	○	○	○
The navigation was consistent when moving between screens. (3)	○	○	○	○	○	○	○	○
The interface of the App allowed me to use all the functions (such as entering information, viewing information) offered by the App. (4)	○	○	○	○	○	○	○	○
Whenever I made a mistake using the App, I could recover easily and quickly. (5)	○	○	○	○	○	○	○	○
I like the interface of the App. (6)	○	○	○	○	○	○	○	○
The information in the App was well organised, so I could easily find the information I needed. (7)	○	○	○	○	○	○	○	○
The App adequately acknowledged and provided information to let me know the progress of my action. (8)	○	○	○	○	○	○	○	○
I feel comfortable using this App in social settings. (9)	○	○	○	○	○	○	○	○

The amount of time involved in using this App has been fitting for me. (10)								
I would use this App again. (11)								
Overall, I am satisfied with this App. (12)								
The App would be useful for my health and well-being. (13)								
The App improved my access to healthcare services. (14)								
The App helped me manage my health effectively. (15)								
This App has all the functions and capabilities I expected it to have. (16)								
I could use the App even when the Internet connection was poor or not available. (17)								
This App provides an acceptable way to receive healthcare services, such as accessing educational materials. (18)								

Page Break

Q84 Is there anything else you would like to tell us about your experience of the App and the care recommendations it made for you?

____

____

____

____

____

End of Block: Overall views about the Tool

Start of Block: About you

Q85 We would now like to ask you a few questions about yourself. This is so we can make sure we have heard the views of a wide range of women from different backgrounds.

Q86 Please tell us the age group you belong to.

- under 16 (1)
- 16 to 19 (2)
- 20 to 24 (3)
- 25 to 29 (4)
- 30 to 34 (5)
- 35 to 39 (6)
- 40 to 44 (7)
- 45 or over (8)
- Would rather not say (9)

Page Break

Q87 Which of the following most closely matches your Ethnicity

- Asian or Asian British (1)
- Black or Black British (2)
- Mixed (3)
- Other Ethnic Groups (4)
- White (5)
- Would rather not say (6)

Display This Question:

If Q87 = Asian or Asian British

Q88 Asian or Asian British - Ethnic Group sub-category

- ○ Indian (1)
- ○ Pakistani (2)
- ○ Bangladeshi (3)
- ○ Any other Asian background (4)
- ○ Would rather not say (5)

Display This Question:

If Q87 = Black or Black British

Q89 Black or Black British - Ethnic Group sub-category

- ○ Caribbean (1)
- ○ African (2)
- ○ Any other Black background (3)
- ○ Would rather not say (4)

Display This Question:

If Q87 = Mixed

Q90 Mixed - Ethnic Group sub-category

- ○ White and Black Caribbean (1)
- ○ White and Black African (2)
- ○ White and Asian (3)
- ○ Any other mixed background (4)
- ○ Would rather not say (5)

Display This Question:

If Q87 = Other Ethnic Groups

Q91 Other - Ethnic Group sub-category

- ○ Chinese (1)
- ○ Any other ethnic group (2)
- ○ Would rather not say (3)

Display This Question:

If Q87 = White

Q92 White - Ethnic Group sub-category

- ○ British (1)
- ○ Irish (2)
- ○ Any other White background (3)
- ○ Would rather not say (4)

Page Break

*

Q93 What is your postcode?   (If you would prefer not to answer, please leave blank).

____

Page Break

Q94 What is your preferred language?   (If you would prefer not to answer, please leave blank).

____

End of Block: About you

Start of Block: Final page

Q95 Thank you for taking part in this survey. The information you have provided will help us to improve the Tommy's App (Pathway) for women in the future.

We also want to talk to some women in more detail about their experience of the Tommy's App and how it may have influenced the pregnancy care they received. We will select 40 women from different backgrounds with different experiences so we can make sure we hear what a range of women feel about the App. These women will be invited to talk to us through one-to-one interviews. We are also arranging group discussions (focus groups) and will invite 5-8 women to take part in one of two focus groups at each hospital.

If you would be happy for us to contact you with more details, please indicate here and enter your name and email address below. (*These details will only be used for the purpose of contacting you with more information about this study*).

Q96 I am happy to be contacted, if selected, with more information about taking part in an interview.

- ○ Yes, I am happy to be contacted if I am selected for interview or focus group. (1)
- ○ No, I would rather not be contacted about an interview or focus group. (2)

Display This Question:

If Q96 = Yes, I am happy to be contacted if I am selected for interview.

Q97 So we can contact you if you are selected for interview or focus group, please enter your name here:

____

Display This Question:

If Q96 = Yes, I am happy to be contacted if I am selected for interview.

*

Q98 ...and your email address here:

____

Page Break

Q99 Finally, if you would like to be entered into our thank you draw for a £100 shopping voucher, please indicate below. (*These details will only be used for the purpose of entering you into the draw*).

Q100 I would like to be entered into the £100 draw.

- ○ Yes, I would like to be entered into the draw. (1)
- ○ No, I would rather not be entered into the draw. (2)

Display This Question:

If Q100 = Yes, I would like to be entered into the draw.

Q101 So we can contact you if you win the £100 draw, please enter your name here:

____

Display This Question:

If Q100 = Yes, I would like to be entered into the draw.

*

Q102 and your email address here:

____

End of Block: Final page
